# Rapid Cue-Specific Remodeling of the Nascent Axonal Proteome

**DOI:** 10.1016/j.neuron.2018.06.004

**Published:** 2018-07-11

**Authors:** Roberta Cagnetta, Christian K. Frese, Toshiaki Shigeoka, Jeroen Krijgsveld, Christine E. Holt

**Affiliations:** 1 Department of Physiology Development and Neuroscience, Downing Street, University of Cambridge, Cambridge CB2 3DY, UK; 2 European Molecular Biology Laboratory (EMBL), Meyerhofstr. 1, Heidelberg 69117, Germany; 3 German Cancer Research Center (DKFZ), Im Neuenheimer Feld 581, Heidelberg 69120, Germany; 4 CECAD Research Center, University of Cologne, Joseph-Stelzmann-Str. 26, Cologne 50931, Germany; 5 Excellence Cluster CellNetworks, University of Heidelberg, Im Neuenheimer Feld 581, Heidelberg 69120, Germany

**Keywords:** axon, retinal ganglion cell, pSILAC-SP3, proteomics, extrinsic cues, axon guidance, chemotropic response, growth cone, local protein synthesis, neural wiring

## Abstract

Axonal protein synthesis and degradation are rapidly regulated by extrinsic signals during neural wiring, but the full landscape of proteomic changes remains unknown due to limitations in axon sampling and sensitivity. By combining pulsed stable isotope labeling of amino acids in cell culture with single-pot solid-phase-enhanced sample preparation, we characterized the nascent proteome of isolated retinal axons on an unparalleled rapid timescale (5 min). Our analysis detects 350 basally translated axonal proteins on average, including several linked to neurological disease. Axons stimulated by different cues (Netrin-1, BDNF, Sema3A) show distinct signatures with more than 100 different nascent protein species up- or downregulated within the first 5 min followed by further dynamic remodeling. Switching repulsion to attraction triggers opposite regulation of a subset of common nascent proteins. Our findings thus reveal the rapid remodeling of the axonal proteomic landscape by extrinsic cues and uncover a logic underlying attraction versus repulsion.

## Highlights

• pSILAC-SP3 reveals the newly synthesized axonal proteome within minutes
• Extrinsic cues rapidly up- and downregulate large subsets of nascent proteins
• Different repulsive cues generate distinct proteomic signatures
• Switch from repulsion to attraction elicits opposite changes in nascent proteome

## Graphical Abstract

**Figure:**
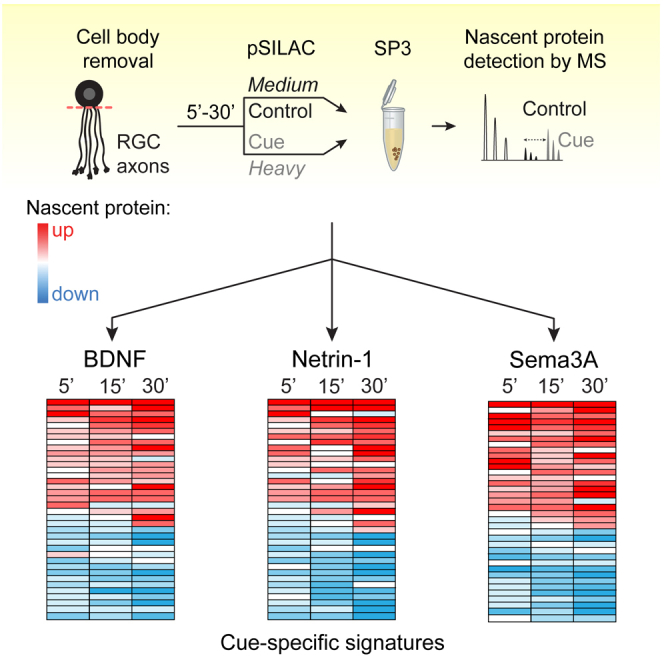

## Teaser

Cagnetta et al. establish a highly sensitive proteomics approach to characterize the nascent proteome of a subcellular compartment (axon) on a rapid timescale (minutes). Remarkably, different extrinsic cues trigger distinct signatures of up- and downregulated nascent protein changes within 5 min.

## Introduction

The generation of neuronal networks requires neurons to connect to their synaptic partners with precision. This process is mediated through axon navigation, guided by the growth cone, which senses growth and guidance factors and transduces these signals to regulate the speed and direction of axon extension, often long distances away from the soma. Growing axons contain complex transcriptomes (Zivraj et al., 2010), and the regulation of local translation and protein degradation allows the rapid and dynamic control of the axonal proteome in response to extracellular stimuli (Campbell and Holt, 2001, Deglincerti et al., 2015). Previous studies have focused on only a handful of candidate transcripts regulated by guidance cues, even though more than 1,000 different mRNAs are known to reside in axons (Leung et al., 2006, Piper et al., 2006, Yao et al., 2006, Bellon et al., 2017, Vidaki et al., 2017, Lepelletier et al., 2017, Wu et al., 2005, Villarin et al., 2016, Hengst et al., 2009, Jain and Welshhans, 2016, Zivraj et al., 2010). It is not known whether cues elicit large-scale translational changes across the axonal transcriptome or whether different guidance molecules trigger the translation of different subsets of mRNAs. A recent genome-wide analysis on the ribosome-associated mRNAs revealed a complex and dynamically regulated translatome in retinal axons *in vivo* (Shigeoka et al., 2016). This approach, however, did not provide information to distinguish the cue-induced translatome from the basal translatome and did not address the question of cue-specific up- and downregulation of newly synthesized proteins (NSPs). These questions require an unbiased analysis of the cue-induced nascent proteome in the axonal compartment over a short timescale.

The characterization and quantification of the nascent proteome of whole cells or tissue samples by metabolic labeling methods, such as pulsed stable isotope labeling of amino acids in cell culture (pSILAC) or bio-orthogonal non-canonical amino acid tagging (BONCAT), are straightforward due to sample abundance and typical labeling periods of hours (Schwanhäusser et al., 2009, Dieterich et al., 2006, Schanzenbächer et al., 2016). However, a systematic analysis of the nascent proteome from very low sample amounts of a subcellular compartment, uncontaminated by the soma, over a short timescale (<20 min) remains a major challenge (Eichelbaum and Krijgsveld, 2014). To overcome these challenges, we applied pSILAC in a compartmentalized chamber in combination with ultrasensitive sample preparation technology, termed single-pot solid-phase-enhanced sample preparation (SP3) (Hughes et al., 2014). This enabled us to characterize and quantify the system-wide proteome dynamics in response to specific guidance cues from tiny amounts (≤2 μg) of somaless retinal axons on a timescale of minutes. Further, we used this unbiased highly sensitive approach to investigate the axonal proteomic changes that characterize the switch between repulsive and attractive chemotropic responses.

Our results revealed that hundreds of proteins, encompassing a wide range of cellular functions, are rapidly translated within the axonal compartment in basal conditions and in response to guidance cues. Comparative analysis of cue-induced newly synthesized proteomes revealed more than 100 nascent protein species regulated within just 5 min, some distinctly associated with specific guidance cues and some common to all cues. When cue-induced responses are switched from repulsion to attraction, a fraction of a common subset of NSPs undergo opposite regulation. Collectively, our data show rapid nascent proteome remodeling in response to guidance molecules and uncover subsets of opposite proteomic changes involved in the switch between repulsive and attractive chemotropic responses.

## Results

### Ultrasensitive Proteomics Detects Rapid Changes in the Axonal Nascent Proteome

**Figure 1:**
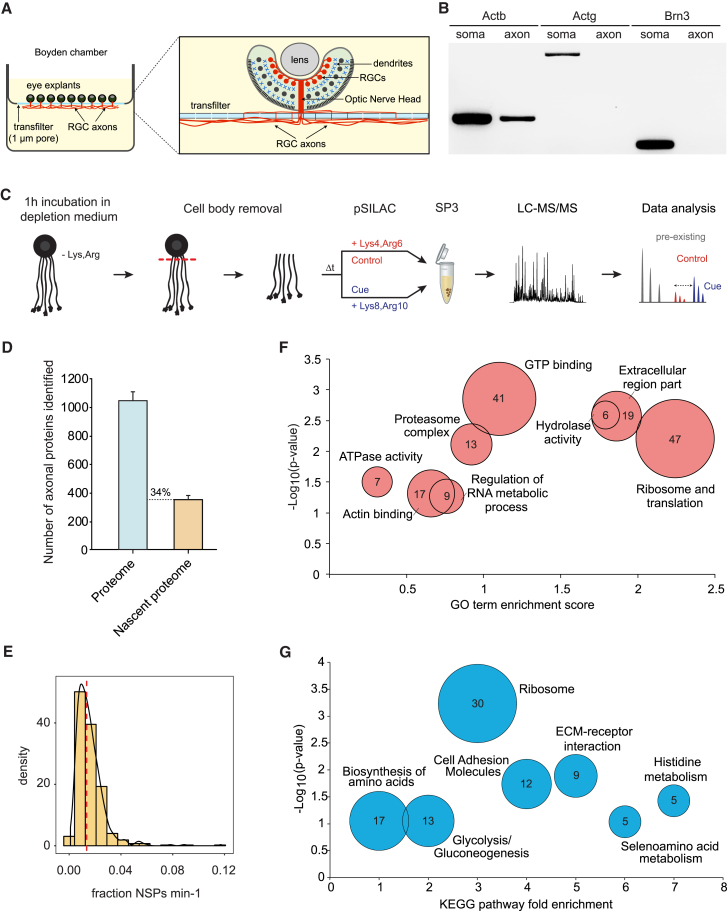
pSILAC-SP3 Detects the Axonal Newly Synthesized Proteome within 5 min (A) Intact whole-eye primordia were cultured in compartmentalized Boyden chambers. Only the axons of RGCs exit the eye—via optic nerve head (ONH)—and extend through the 1 μm pores to grow on the laminin-coated underside of the transfilter. Cell bodies and dendrites remain in the eye on the upper surface and are removed immediately prior the experiment, leaving pure somaless axons. (B) RT-PCR confirms the purity of the axonal compartment. Actb (positive control; Leung et al., 2006), but not Actg or Brn3 (negative controls; Willis and Twiss, 2011, Yoon et al., 2012), mRNAs were detected. (C) Schematic representation of the pSILAC-SP3 methodology applied to somaless retinal axons. Axons are exposed to vehicle or cue in pSILAC medium containing either “medium” or “heavy” isotope-coded Arg and Lys. (D) Proteins identified in axons derived from 100 eye explants and NSPs identified in axons after 5 min of pSILAC. Error bars, SEM. (E) Density distribution of basal NSPs per minute relative to total protein amount. Red line indicates the median. (F) Enriched GO terms in the biological process, molecular function, and cellular composition categories for constitutive axonal NSPs (p < 0.1). (G) Enriched KEGG pathways for constitutive axonal NSPs (p < 0.1). Circle size and numbers indicate NSP counts. See also Figure S1.

First, we tested whether the SP3 approach (Hughes et al., 2014) was sufficiently sensitive for detecting the nascent proteome of axons of a single cell type within 5 min in basal (non-stimulated) conditions. To obtain isolated axons, we cultured intact whole-eye primordia from *Xenopus laevis* embryos in compartmentalized transfilter chambers (Figure 1A) (Willis and Twiss, 2011). Explanted whole eyes maintain their anatomical integrity in culture, and only the axons of retinal ganglion cells (RGCs) exit the eye to grow through the 1 μm pores of the transfilter onto the lower surface (Figure 1A). Immunostaining of eye sections with specific markers verified the exclusive presence of RGC axons exiting the eye (Figures S1A–S1C) and the absence of nuclei and dendrites (Figures S1D and S1E) on the lower surface of the transfilter. Axonal purity was further demonstrated by RT-PCR, showing the presence of β-actin mRNA and of the axonal marker microtubule-associated protein Tau (Mapt) mRNA (Figure 1B; Figure S1F) (Leung et al., 2006, Litman et al., 1993) and the absence of somal and dendritic mRNAs in the axonal material collected from the transfilter (Figure 1B; Figure S1F).

Given the limited availability of axonal material, we assessed the detection sensitivity of the SP3 protocol on axon samples derived from 100 eye explants (≤2 μg protein typical yield) (Hughes et al., 2014). SP3-MS (mass spectrometry) analysis identified more than 1,000 proteins (Figure 1D; Table S3). In line with our results on axonal purity (Figures S1D–S1F), the RGC marker γ-synuclein was detected in the axonal proteome, whereas dendrite markers were absent (Table S3). To investigate the newly synthesized proteome, we incubated retinal cultures for 1 hr in medium depleted of lysine and arginine. Subsequently, the cell bodies were eliminated by eye removal from the upper surface of the transfilter, and the somaless axons on the lower transfilter surface were incubated for 5 min with “Medium” (M) isotope-coded amino acids (Lys4, Arg6). After lysis, the pSILAC axon samples were processed by SP3 prior to MS analysis (Figure 1C). pSILAC-SP3 detected an average of 350 NSPs that incorporated the labeled amino acids under basal conditions following a 5 min pulse of SILAC in a ≤2 μg axonal sample (Figure 1D; Table S3).

The distribution of the M label incorporation rate in NSPs relative to total protein, calculated as (M/(M+L))/5 min, where “Medium” represents the basally NSPs and “Light” (L) indicates the pre-existing proteins, showed that protein synthesis occurs at a basal level in non-stimulated axons and that signals, although low, were consistently detectable (median = 1.4% of the total signal; Figure 1E). Samples were analyzed in biological triplicate to assess reproducibility, which yielded an average Pearson’s correlation coefficient (PCC) of 0.6 between replicates (Figure S1G), higher than the PCC derived from 20 min BONCAT-pSILAC previously carried out on ∼10^7^ RAW 264.7 macrophages (PCC = −0.11; Eichelbaum and Krijgsveld, 2014). These data indicate that approximately one-third of the axonal proteome is synthesized locally (Figure 1D). A positive correlation was found between the abundance of pre-existing proteins and NSPs (Figure S1H), indicating borderline detection of *de novo* synthesis for low-abundance proteins due to the sample scarcity and short timescale. The ratios between newly synthesized and pre-existing proteins correlated negatively with the abundance of the pre-existing proteins (PCC = −0.5; Figure S1I), suggesting that the average basal translation rate is relatively constant for the majority of the proteins.

To gain insight into the potential function of basal protein synthesis in developing axons, we conducted functional Gene Ontology (GO) enrichment analysis. “ribosome and translation” was the most-enriched category (Figure 1F), consistent with evidence from the *in vivo* mouse axonal translatome (Shigeoka et al., 2016). The “proteasome complex” was also significantly enriched (Figure 1F), in line with the *in vivo* mouse axonal translatome and with previous work showing that ubiquitin-proteasome system (UPS) components are abundant in growth cones and that axonally synthesized proteins undergo a particularly high degree of UPS-dependent turnover (Deglincerti et al., 2015). A further highly enriched cluster was the “extracellular region part,” containing extracellular matrix (ECM) proteins. Of note, these categories were much more enriched than the “actin binding” one (Figure 1F). Similar to the GO analysis, Kyoto Encyclopedia of Genes and Genomes (KEGG) pathway analysis revealed significant enrichment in “ribosome,” “cell adhesion molecules,” and “ECM-receptor interaction” pathways (Figure 1G). KEGG analysis also highlighted several metabolic pathways linked to amino acid synthesis, such as “histidine metabolism” and “glycolysis,” suggesting that the axon is equipped with its own biosynthetic processes and energy supply (Figure 1G; Figure S1L). Several of the metabolic NSPs belonged to the “mitochondrion” and “mitochondrial matrix” GO categories (Figure S1L). Categories related to stress and apoptosis were absent, consistent with evidence showing that axons deprived of their cell bodies do not exhibit anatomical signs of degeneration up to ∼3 hr (Harris et al., 1987).

The results demonstrate that NSPs can be detected in extremely small quantities of subcellular material within minutes by pSILAC-SP3. Furthermore, they show that developing axons basally synthesize proteins encompassing a wide range of cellular functions, suggesting the capacity to locally replenish metabolic proteins and machineries subject to high protein turnover.

### Guidance Cues Trigger Rapid Wide-Scale Remodeling of the Nascent Axonal Proteome

To investigate whether guidance cues induce a proteome-wide response and whether it changes over time, we carried out pSILAC-SP3 on somaless axons stimulated with three different cues present along the visual pathway (brain-derived neurotrophic factor [BDNF], Netrin-1, and Semaphorin3A [Sema3A]), together with “heavy” (H) isotope labels (Lys8, Arg10), for 5, 15, and 30 min. Control somaless axon samples were incubated simultaneously with M isotope labels (Lys4, Arg6). Subsequently, in each independent replicate, control and cue-stimulated samples sharing the same pulse duration were lysed, pooled, and processed through SP3 (Figure 1C). To obtain ∼2 μg of axonal material, we explanted 100 eye primordia per transfilter, yielding <5,000 axons, and cultured more than 1,800 eyes for each single replicate.

**Figure 2:**
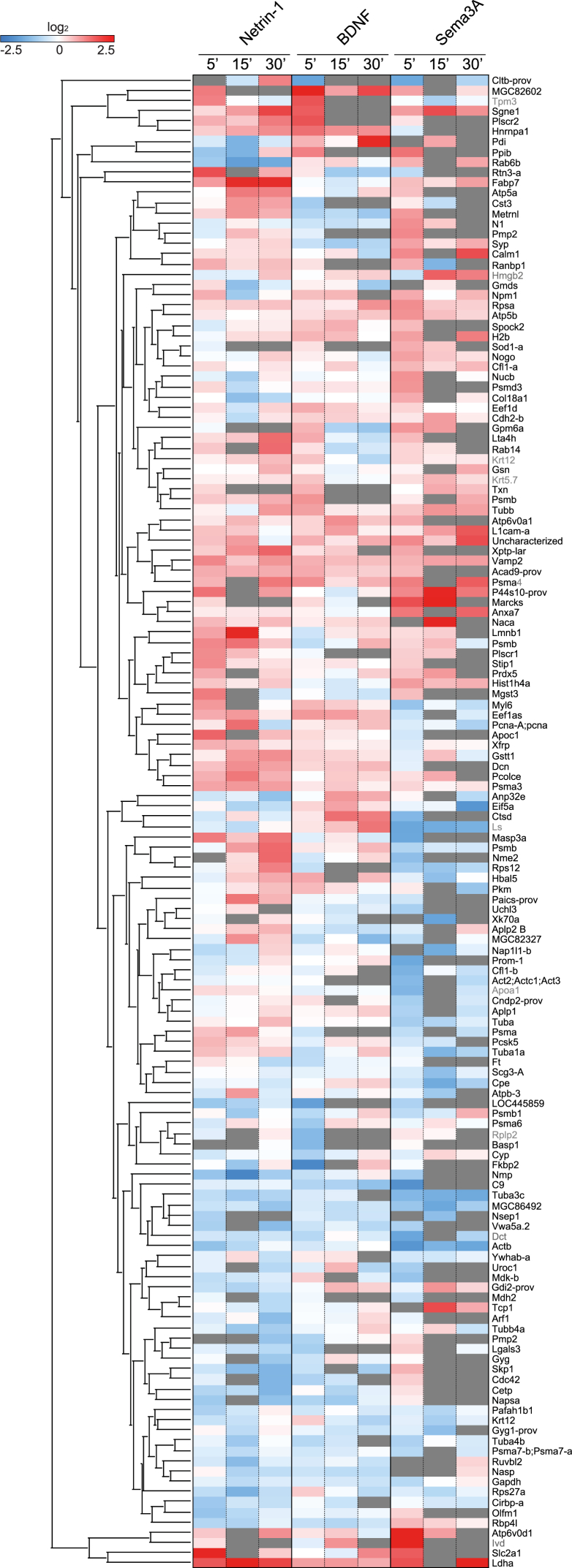
Guidance Molecules Trigger Rapid and Wide-Scale Up/Down Remodeling of the Nascent Axonal Proteome Hierarchical clustering of cue-induced NSP changes (log_2_(H/M)) at different time points (5′, 15′, 30′) derived from ≥3 independent biological replicates per each cue stimulation (derived from 5, 5, and 3 independent biological replicates for stimulation with BDNF, Netrin-1, and Sema3A, respectively). Red indicates upregulation, blue indicates downregulation, and dark gray indicates NSP quantified in <50% of biological replicates. Proteins not annotated in *Xenopus laevis* were blasted against *Xenopus tropicalis* (identity ≥ 90%, indicated in gray). See also Figure S2.

The MS analysis revealed up to 500 quantified NSP changes per cue stimulation undergoing a similar trend across the biological replicates (meeting the requirements (1) quantified in >50% of biological replicates, (2) standard deviation (SD) ≤ 1, (3) average log_2_(H/M) > |0.3|) and up to 166 significant NSP changes per cue stimulation, thus indicating that guidance cues cause wide-scale remodeling of the nascent proteome (Figure 2; Table S3). This large set of changes was reproducibly detected after just 5 min of cue stimulation (up to 108 significant NSP changes). Whereas previous studies have mainly shown that some mRNAs can be translated in response to cue stimulation, our data revealed that, in addition to upregulation, several NSPs undergo cue-induced downregulation (Figure 2), thus indicating tight control of the axonal nascent proteome in both directions. As expected, given the limiting rate of protein synthesis, the NSPs upregulated in response to cues at the 5 min time point are shifted slightly toward a smaller size with respect to the axonal proteome size distribution (Figure S2A).

We next asked how cue-induced NSP changes correlate with the abundance of their local transcripts by comparing the mRNA levels in retinal growth cones of *Xenopus* stage 32 (Zivraj et al., 2010) with the abundance of repulsive cue-induced heavy-labeled NSPs. This yielded a significant but low positive correlation (ρ = 0.33; Figure S2B), as seen in other systems (Gygi et al., 1999, Rajasundaram et al., 2014). The results suggest that cue-induced NSP changes, supported to some degree by local transcriptome levels, rapidly remodel the axonal nascent proteome on a wide scale in both up and down directions.

### Validation of the Axonal Nascent Proteome

**Figure 3:**
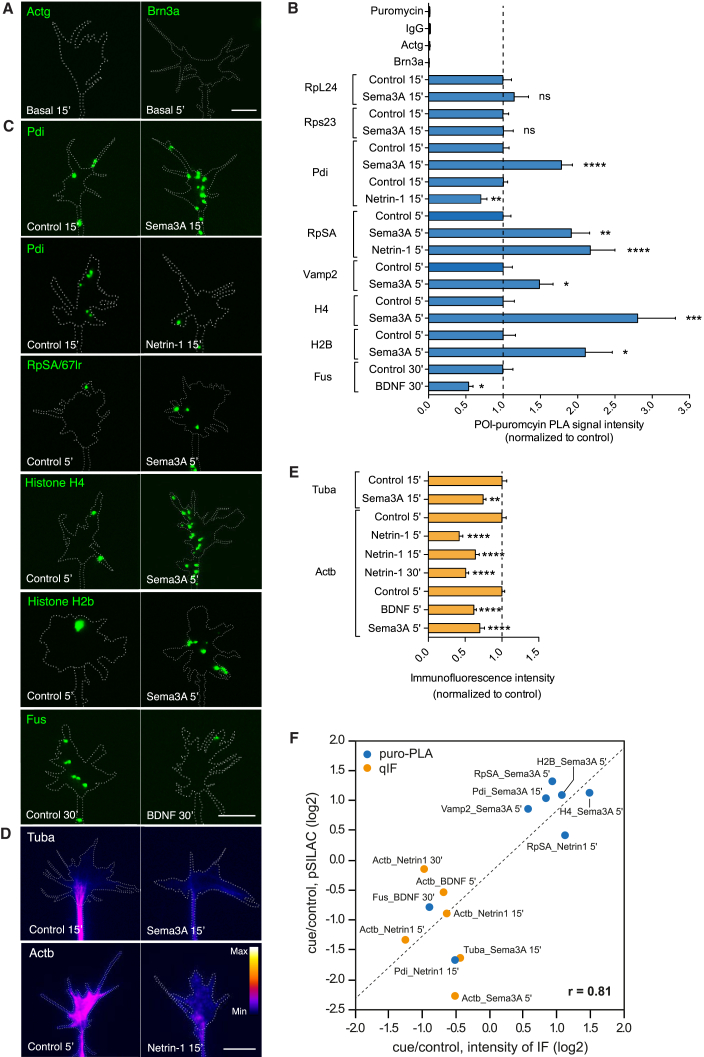
Validation of the pSILAC-SP3 Approach in Axonal Growth Cones (A) Images of puro-PLA for negative controls (Figure 1B). (B and C) Puro-PLA quantification (B) and representative images (C) to validate NSPs across different functional categories, conditions, and time points (Table S1; Mann-Whitney test and one-way ANOVA with Bonferroni’s multiple comparisons test). (D and E) IF representative images (D) and quantification (E) to validate NSPs across different functional categories, conditions, and time points (Table S1; Mann-Whitney test and one-way ANOVA with Bonferroni’s multiple comparisons test). (F) Direct comparison of pSILAC and IF-derived detection of NSPs reveals excellent correlation with r = 0.81 (r_puro-PLA_ = 0.87; r_qIF_ = −0.41). Error bars, SEM. Scale bars, 5 μm. See also Figure S3.

To validate the pSILAC-SP3 data, we used two orthogonal methods: (1) puromycylation combined with proximity ligation assay (puro-PLA) to quantify protein synthesis (Figures 3A–3C; Figure S3A; Table S1) (tom Dieck et al., 2015) and (2) quantitative immunofluorescence (qIF) to measure the overall total protein level (Figures 3D and 3E; Figure S3A; Table S1). For puro-PLA, the use of one single antibody and rabbit IgG served as two independent negative controls, confirming the absence of a non-specific signal (Figure 3B; Figure S3A). Sixteen NSP changes were chosen from the proteomic dataset for validation, representing proteins across different functional categories, in stimulated and non-stimulated conditions, after various pulses of SILAC (5, 15, or 30 min) and exhibiting different average log_2_ ratios (Table S1). We confirmed the two negative controls (Figures 3A and 3B) whose transcripts were not detected in axons (Figure 1B) and successfully validated 15 out of 16 NSP changes (Figures 3B–3E; Figure S3A). The majority of the mRNAs corresponding to the validated NSPs were previously detected in axons (Table S1). The pSILAC and fluorescence-based “cue/control condition” ratios showed a strong positive correlation (PCC = 0.81) (Figure 3F). The correlation was higher for the puro-PLA data than the immunofluorescence (IF) data, which was expected due to the higher degree of specificity of the puro-PLA method that detects only the NSPs and not total protein. For example, at 5 min, both pSILAC and puro-PLA revealed that Sema3A upregulates histone H4 translation (Figures 3B and 3C; Table S1), whereas the total protein amount of H4 does not change at 5 min and significantly decreases following 15 min Sema3A stimulation (Figure S3B). This decrease is blocked by Ciliobrevin A, a dynein inhibitor that prevents retrograde transport (Figure S3B) (Firestone et al., 2012), suggesting that H4 may be transported back to the nucleus after being locally translated in response to Sema3A. This indicates that the amount of total protein does not necessarily correlate with the amount of locally synthesized protein and illustrates a further dynamic complexity in the proteomic response to cues.

It should be noted that while pSILAC-SP3 was carried out on axons plus growth cones (Figure 1C), the validation above was carried out on single growth cones as these are easily identifiable by their expanded morphology, aiding accurate quantification of the immunofluorescence signal per unit area, whereas retinal axons often fasciculate together in bundles, making quantitative measurement more challenging. To test the validity of growth cone sampling on axons, we selected one of the growth-cone-validated NSP changes (puro-Pdi PLA in response to 15 min Sema3A stimulation; Figures 3B, 3C, and 3F) and conducted qIF on the axon shaft (distal 20 μm segment). The results correlated with both the growth cone qIF values and the pSILAC (Figures S3C and S3D; Figure 3F; Table S1), indicating consistency between the two types of sampling analyses (growth cone versus axon).

We also interrogated the pSILAC datasets for proteins previously reported to be axonally translated in basal conditions or in response to the same cues and stimulation time used in our experiments. The analysis confirmed the pSILAC-SP3 detection of the majority of protein changes (8/10), including some from axons of other species and types of neurons (Table S2). The 2/10 protein changes not detected by pSILAC-SP3 were previously reported in mouse cortical and hippocampal growth cones and identified in the retinal axonal translatome (Table S2) (Shigeoka et al., 2016). This suggests that either the pSILAC-SP3 approach has limited sensitivity due to the paucity of axonal sample and the rapid timescale or that not all responses to cues are conserved among different species.

Finally, we compared the pSILAC-SP3 outcome in response to cues versus the mRNAs bound *in vivo* to ribosomes in the RiboTag mouse RGC axons at three developmental stages: embryonic day 17.5 (E17.5) (axon elongation), postnatal day 0.5 (P0.5) (axon branching), and P7.5 (pruning) (Shigeoka et al., 2016). Comparative analysis revealed an overlap at stages E17.5 and P0.5 (Figure S3E), consistent with previous evidence showing a physiological relevance for the cues investigated and local translation in axon elongation, branching, and synaptogenesis (Hengst et al., 2009, Wong et al., 2017, Campbell et al., 2001, Cohen-Cory and Fraser, 1995, Manitt et al., 2009, Lyles et al., 2006, Aguado et al., 2003). The abundance of the NSPs showed a positive correlation with the RiboTag data (Figure S3E), although this was unsurprisingly low given the difference between the *in vitro* and the *in vivo* context (e.g., axons experience multiple cues simultaneously in the tectum) and the different species.

### Guidance Cues Induce a Temporally Dynamic Newly Synthesized Proteome

**Figure 4:**
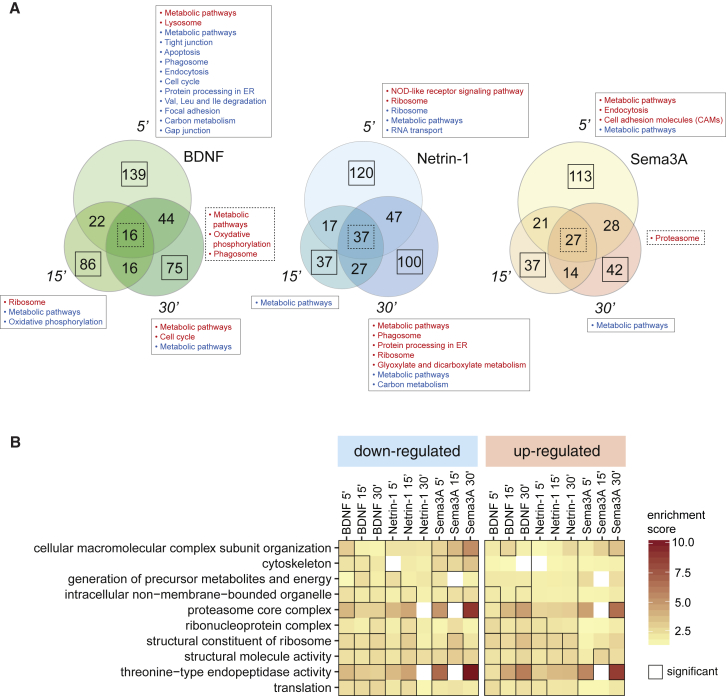
Nascent Axonal Proteome Changes Dynamically over the Duration of Cue Stimulation (A) Overlap of the NSP changes among different times of stimulation in response to each cue. Both common (i.e., the same NSP undergoes similar directional change with log_2_(H/M) ratio > |0.3|) and different (i.e., a new NSP exhibits change with log_2_(H/M) ratio > |0.3| or the same NSP undergoes opposite directional change) NSP changes were detected among different cue stimulation periods. Rectangles with solid lines outline KEGG pathway analysis for the NSP changes unique for each time point, and rectangles with dashed lines outline KEGG pathway analysis for the NSP changes constant among different time points (cutoff ≥ 3 proteins per pathway). Red indicates upregulated pathways, and blue indicates downregulated pathways. (B) Enriched GO terms in the biological process, molecular function, and cellular composition categories of selected categories (category count > 15; for complete table, see Figure S4). Rectangles indicate significantly enriched GO terms (p < 0.05). See also Figure S4.

Next, we asked whether the newly synthesized proteome is dynamic over the 30 min period of stimulation. Both common and different NSP changes were detected among different cue stimulation periods (5 min, 15 min, 30 min; Figure 4A). For example, several vacuolar ATPase subunits belonging to the metabolic pathways were upregulated in response to BDNF over the whole stimulation time (Figure 4A). Several proteins underwent translational change at 5 min, but not 15 and 30 min, suggesting that these proteins might subsequently undergo homeostatic regulation, UPS-mediated degradation, retrograde transport, or secretion. For example, several proteins involved in endocytosis (e.g., Rab11b) were uniquely upregulated after 5 min Sema3A stimulation but not at later time points, possibly due to an immediate burst in synthesis followed by retrograde transport (Figure 4A) (Zylbersztejn et al., 2012). Similarly, the Netrin-1 axon guidance receptor Kazald1 was the most upregulated NSP within 5 min Netrin-1 stimulation but was not detected at later time points, suggesting an autocrine mechanism underlying the rapid amplification of Netrin-1 signaling (Cheng et al., 2011). Other NSPs were regulated only at later time points (e.g., some ribosomal proteins) (Figure 4A). Similarly, functional enrichment analysis revealed GO clusters that were constant (e.g., “cytoskeleton”) and differentially enriched (e.g., “cellular macromolecular complex subunit organization”) along the time course of one cue stimulation (Figure 4B; Figure S4). These data indicate that part of the cue-induced axonal nascent proteome changes dynamically during the period of stimulation.

### Different Guidance Molecules Induce Cue-Specific Signatures

**Figure 5:**
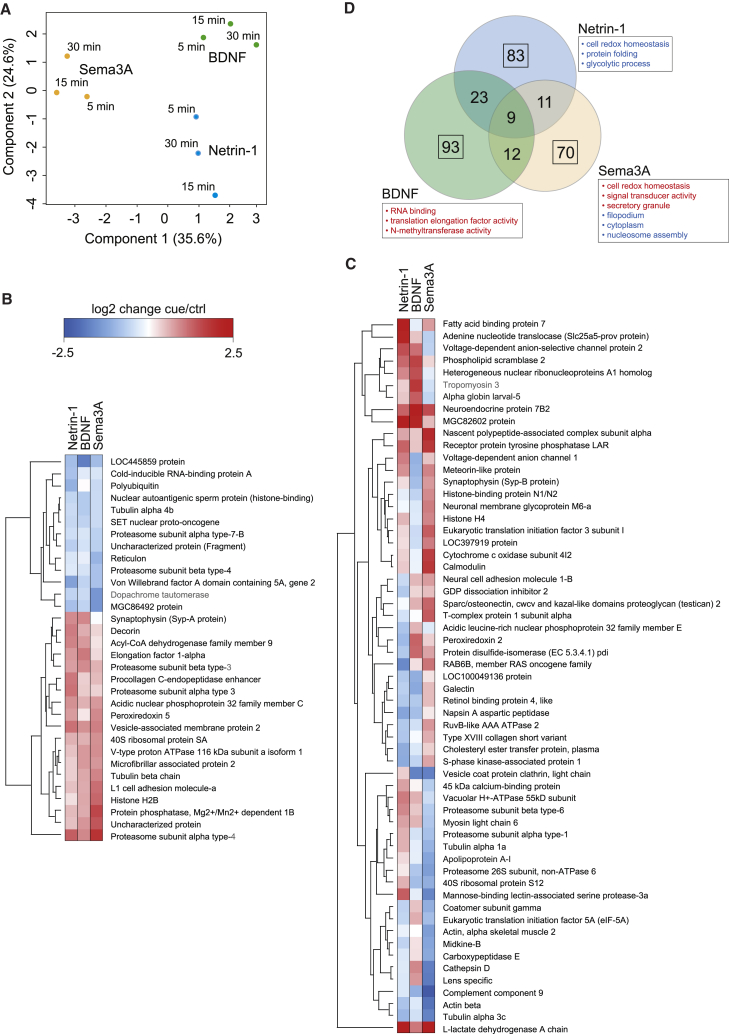
Different Guidance Molecules Induce Both Common and Distinct NSP Signatures (A) Principal component analysis (PCA) based on the NSP changes identified in response to the three cue stimulations and time points. (B) Hierarchical clustering of the NSP changes (averaged over the three time points) exhibiting similarity (SD < 0.5) among different cue stimulations. (C) Hierarchical clustering of the NSP changes (averaged over the three time points) exhibiting diverging behavior (SD > 0.5) among different cue stimulations. Proteins not annotated in *Xenopus laevis* were blasted against *Xenopus tropicalis* (identity ≥ 90%, indicated in gray). (D) Overlap of the three cue-induced NSP changes (averaged over the three time points). Rectangles outline the NSP changes unique per cue stimulation and their enriched GO terms in the biological process, molecular function, and cellular composition categories (p < 0.1).

To discover whether different guidance cues induce the same or different sets of NSP changes, we compared the nascent proteomes of axons in response to the three cues—BDNF, Netrin-1, and Sema3A—at three different time points. Principal component analysis (PCA) showed that the factor contributing the most to the total variation is the type of cue (Figure 5A). The stimulation time also contributed to the total variation (consistent with our previous findings; Figures 4A and 4B), but the three time points for each cue stimulation clustered together, indicating that they are coordinately controlled by a specific cue signaling pathway (Figure 5A).

To quantify and identify the different and common NSP changes in response to the three cue stimulations, we first filtered our datasets by averaging the three time points. We then partitioned proteins with SD < 0.5 (“common” among different cue stimulations; Figure 5B) and >0.5 (“different” among different cue stimulations; Figure 5C). NSPs such as cytoskeletal proteins (e.g., β-actin and several α- and β-tubulin isotypes), cell adhesion proteins (e.g., L1cam-a), proteins involved in endo- and exocytosis (e.g., Vamp2), “nuclear” proteins (e.g., histone H2b), and several proteasomal subunits were similarly regulated by all three cues (Figures 5B and 5C). As these cues all act as chemorepellents in our culture conditions (10 μg/mL laminin substrate; Höpker et al., 1999, Campbell and Holt, 2001), the shared NSP changes may underlie a common chemorepulsive mechanism.

In addition to these common NSPs, the different cues also elicited distinct changes (Figure 5C). For example, the RNA-binding protein (RBP) Hnrnpa1 was upregulated in response to Netrin-1 and BDNF, but not Sema3A, in line with previous evidence showing that BDNF stimulation increases Hnrnpa1 and its association with specific mRNAs (Zielinski et al., 2006). Histone H4 was upregulated in response to Sema3A and Netrin-1 but not in response to BDNF (Figure 5C). Several proteasomal and ribosomal proteins, as well as proteins involved in neurite and filopodia adhesion, outgrowth, and motility, also exhibited differential regulation in response to distinct cues (Figure 5C). For example, Tuba1a was upregulated in response to Netrin-1 but downregulated in response to Sema3A (Figure 5C).

Next, we analyzed the NSP changes unique to each cue and found 83 for Netrin, 93 for BDNF, and 70 for Sema3A (Figure 5D). Functional enrichment analysis revealed that, for example, BDNF, but neither Netrin-1 nor Sema3A, upregulated the “RNA binding” group comprising several RBPs involved in mRNA transport and splicing and various translational control factors, such as eIF5A (Figure 5D), reminiscent of its role downstream of NGF stimulation in mediating cell survival and neurite outgrowth (Huang et al., 2007). Netrin-1 uniquely downregulated the “cell redox homeostasis” functional cluster whereas Sema3A upregulated it. Sema3A uniquely upregulated GTP binding proteins reported to be involved in growth cone collapse (Figure 5D) (Igarashi et al., 1993). Collectively, the data reveal shared signatures, possibly underlying a common chemorepulsive action, and cue-specific changes in the nascent proteome.

### Repulsive Gradient Elicits Proteomic Changes with Opposite Spatial Polarity

**Figure 6:**
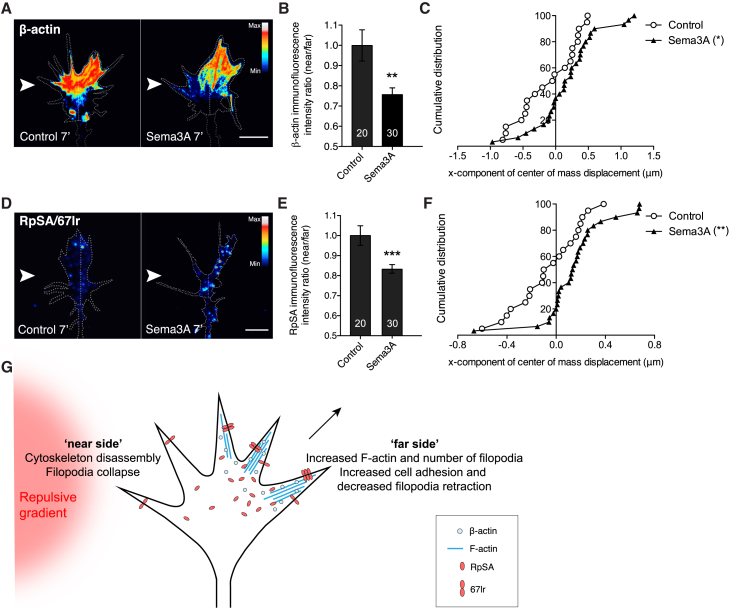
Repulsive Cue Gradient Elicits Proteomic Changes in RpSA and β-actin with Opposite Spatial Polarity within the Growth Cone (A) Growth cone stained for β-actin with a line dividing the near and far sides. Arrow indicates the 90° polarized gradient. (B) Asymmetric decrease of β-actin assessed by the near/far ratio method (unpaired t test). (C) Asymmetric decrease of β-actin assessed by “center of mass” method (unpaired t test). (D) Growth cone stained for RpSA with a line dividing near and far sides. Arrow indicates the 90° polarized gradient. (E) Asymmetric decrease of RpSA assessed by near/far ratio method (unpaired t test). (F) Asymmetric decrease of RpSA assessed by center of mass method (unpaired t test). (G) Repulsive model: β-actin decreases on the near-stimulus side, helping cytoskeleton deconstruction and growth cone collapse, whereas RpSA/67lr increases on the far-stimulus side, thus increasing F-actin and cell adhesion. Error bars, SEM. Scale bars, 5 μm. See also Figure S5.

To explore the biological significance of the cue-induced proteomic changes and their potential role in the chemotropic response, we analyzed NSP localization in growth cones following gradient stimulation. We chose two NSPs belonging to different functional categories and undergoing the same change in response to all three different repulsive cue stimulation: β-actin, which is commonly downregulated, and RpSA (40S ribosomal protein SA), which is commonly upregulated (Figures 2, 5B, and 5C). We reasoned that a repulsive gradient may elicit a polarized decrease in β-actin on the near-stimulus side of the growth cone, leading to polarized (near-side) filopodial collapse and repulsive turning. To test this, a gradient of Sema3A was applied for 7 min at a 90° angle to the growth cone to achieve a steep difference between the “near” and the “far” sides of the growth cone. As predicted, qIF indicated that a Sema3A gradient causes a rapid decrease in β-actin on the near- versus the far-stimulus side (Figures 6A and 6B). We confirmed the asymmetrical localization by comparing the center of mass of β-actin signal intensity between the control and Sema3A conditions, which revealed an equivalent shift away from the Sema3A gradient (Figure 6C).

RpSA was the only ribosomal protein to undergo the same change (increase) in response to all three repulsive cue stimulations (Figures 2 and 5B). When dimerized, RpSA (a.k.a. 67 kDa laminin receptor [67lr]), can play an extra-ribosomal role by functioning as a cell surface receptor for laminin contributing to cell adhesion, cytoskeletal rearrangement, and directional turning (Jamieson et al., 2008, Höpker et al., 1999, Umeda et al., 2008). Selective activation of 67lr with epigallocatechin-3-gallate (EGCG) (Tachibana et al., 2004) caused an increase in the number of filopodia and filamentous-actin (F-actin) (Figures S5A–S5C). Live imaging revealed that this was due to decreased filopodial retraction (Figure S5D), an effect likely due to increased cell adhesion, as suggested by a marked decrease in growth cone speed (Figure S5E). We then asked how RpSA/67lr was distributed spatially across the growth cone in response to a Sema3A gradient, as in the β-actin experiment above. qIF revealed a significant increase in RpSA signal on the far-stimulus side of the growth cone (Figures 6D and 6E). Center-of-mass displacement analysis confirmed a significant shift away from the gradient side (Figure 6F).

The results point toward a general model whereby polarized repulsive stimuli elicit a complex set of spatially compartmentalized proteomic changes that remodel the growth cone and direct turning. Those on the near-stimulus side, for example, promote cytoskeletal disassembly (decrease in β-actin), while those on the far side promote adhesion (increase in 67lr), cytoskeleton assembly (increase in F-actin), and filopodial accumulation, thus helping the growth cone to steer away from the repulsive source (Figure 6G).

### Switching from Repulsion to Attraction Induces Opposite Changes in the Nascent Proteome

**Figure 7:**
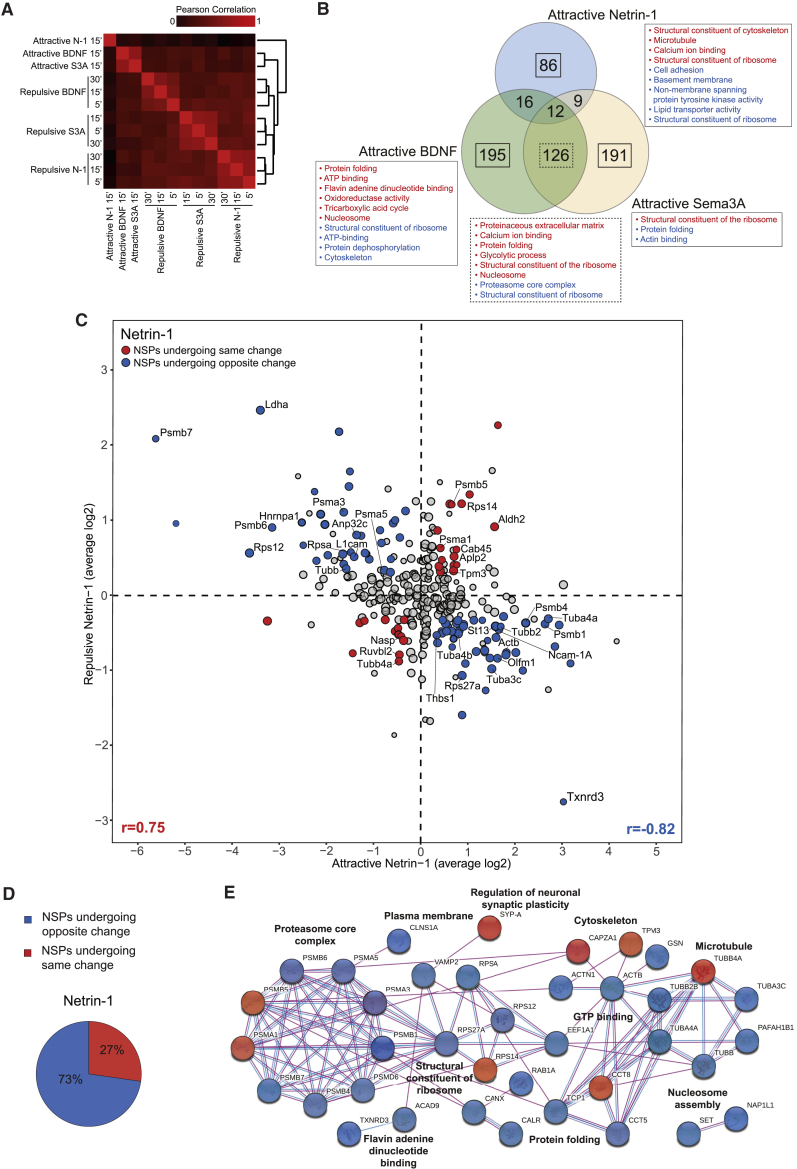
Opposite Regulation of Shared NSPs Underlies Conversion of Netrin-1 Repulsion to Attraction (A) PCC correlation values among the different cue-induced axonal nascent proteomes. (B) Overlap of the three attractive cue-induced NSP changes following 15 min stimulation. Solid rectangles outline the NSP changes unique for each cue stimulation, and dashed rectangles outline the NSP changes in common between Sema3A and BDNF and their related enriched GO terms in the biological process, molecular function, and cellular composition categories (p < 0.1). (C) Plot showing repulsive and attractive Netrin-1 ratios. Circle size correlates with count number, and colored dots indicate commonly regulated NSPs (count > 50%, average ratio > |0.30|). Blue indicates NSPs undergoing opposite change, and red indicates NSPs undergoing the same change. Examples of NSP changes are labeled with protein name (for complete list, see Table S5). (D) Common NSP changes after converting Netrin-1 repulsion into attraction (count > 50%, average ratio > |0.30|). (E) Network-based cluster analysis of the enriched Netrin-1-induced NSP changes and their associated functional classes (p < 0.1). Blue nodes indicate NSPs undergoing opposite change, red nodes indicate NSPs undergoing same change, light blue lines indicate interactions known from databases, and purple lines indicate interactions experimentally determined. Disconnected nodes are not shown (i.e., more NSPs for each enriched functional cluster have been detected). See also Figures S6 and S7.

We next investigated how the newly synthesized axonal proteome changes when a cue is switched from repulsive to attractive. Axons were co-treated with a cue and one of the following pharmacological reagents that switch the directional response of the growth cone from repulsive to attractive: Tautomycin, an inhibitor of protein phosphatase 1, for Netrin-1 (Wen et al., 2004); Sp-cAMP, a membrane-permeable analog of cyclic adenosine monophosphate (cAMP), for BDNF (Song et al., 1997); and 8-Br-cGMP, a membrane-permeable agonist of cyclic guanosine monophosphate (cGMP), for Sema3A (Song et al., 1998). Three not mutually exclusive scenarios were envisaged: switching to attraction induces (1) distinct NSP changes, (2) opposite changes for the same NSPs, and (3) the same NSP changes with opposite spatial distribution within the growth cone. Correlation analysis showed that the repulsive cue-induced nascent proteomes corresponding to the three time points clustered together, whereas they exhibited a PCC close to 0 with respect to the attractive cue-induced nascent proteomes (Figure 7A). This outcome was consistent with the PCA (Figure S6A), thus pointing toward hypotheses 1 and 2.

Similar to the repulsive cue-specific outcome (Figure 5D), the attractive cue-induced nascent proteome analysis revealed that the majority of the NSP changes were cue specific, although BDNF and Sema3A shared a much higher number of NSP changes than Netrin-1 (Figure 7B; Table S4). Pharmacological treatments, it should be noted, may also induce additional effects (e.g., Sp-cAMP can alter mTOR in addition to protein kinase A; Kim et al., 2010). We then compared the attractive cue-induced nascent proteomes to the respective repulsive ones. We found that up to 122 NSPs were commonly regulated between repulsion and attraction (Figure 7C; Figures S6B and S6C; Table S5). In strong support of hypothesis 2, up to 73% of these common subsets (53%, on average, among the three cue stimulations) underwent an opposite change with the switch from repulsion to attraction (Figure 7D; Figure S6D; Table S5) and exhibited a strong negative correlation (PCC = −0.79, on average, among the three cue stimulations; Figure 7C; Figures S6B and S6C). Some NSP changes were cue specific; for example, Olfactomedin 1 (Olfm1) was specifically downregulated in response to repulsive Netrin-1 and upregulated in attractive conditions (Figure 7C), in accord with recent work on mouse dorsal root ganglia showing that Olfm1 is involved in inhibiting growth cone collapse and promoting axon growth (Nakaya et al., 2012). Cortactin (Ctt), reported to promote actin network density, lamellipodial protrusion, and filopodial formation and stability in growth cones, was specifically upregulated in response to attractive BDNF and downregulated in response to repulsive BDNF (Figure S6B) (He et al., 2015). Growth-associated protein-43 (Gap43), whose knockdown causes a failure in commissural axon guidance and reduced levels of F-actin in growth cones (Shen et al., 2002), was specifically upregulated in response to attractive Sema3A and downregulated in response to repulsive Sema3A (Figure S6C). Some NSP changes were shared between common repulsive-attractive subsets of distinct cue stimulations (Figures S6B and S6C). For example, the cell adhesion molecule L1cam, necessary for topographic mapping of retinal axons (Demyanenko and Maness, 2003), was upregulated in response to all three repulsive cues and downregulated in response in all three attractive conditions (Figure 7C; Figures S6B and S6C). Together with our findings of polarized RpSA/67lr synthesis (Figures 5B and 6D–6G), this suggests a generalized cell adhesion model in which adhesion molecules increase on the far-stimulus side in response to a polarized repulsive gradient and decrease on the far-stimulus side in response to an attractive gradient.

Interestingly, 47% of NSP changes, on average, underwent the same change in attractive and repulsive conditions (Figure 7D; Figure S6D), exhibiting a strong correlation (PCC = 0.72, on average, among the three cue stimulations; Figure 7C; Figures S6B and S6C). This is exemplified by the upregulation of many proteins known to underlie the formation of presynaptic terminals and functional synapses (e.g., Cadm1, Ptprd) in response to both repulsive and attractive BDNF (Figure S6B), in accord with previous work showing that BDNF elicits synaptogenesis (Biederer et al., 2002, Takahashi and Craig, 2013, Aguado et al., 2003).

Network-based functional enrichment analysis of the NSPs regulated downstream of each Netrin-1, BDNF, and Sema3A pathway in both attractive and repulsive conditions revealed that several NSP changes undergoing the same or opposite regulation belong to classes of proteins with related biological function (Figure 7E; Figures S6E and S6F), indicating that functionally coherent sets of mRNAs are differentially translationally regulated. A network-based overview of the functionally enriched groups regulated by the three repulsive-attractive cues showed that several of these functional clusters are conserved across different cue stimulations, although the particular proteins regulated can be cue specific (Figure S7). Collectively, the results strengthen the finding that different cues induce distinct proteomic signatures. Furthermore, they show that guidance molecules coordinate functionally coherent sets of NSPs and switch between repulsion and attraction by inducing distinct NSP changes and by bidirectionally regulating common NSPs.

### Neurological Disorder-Associated NSPs Suggest Novel Connections between Local Translation and Disease

In light of recent work showing that constitutive translation of Lamin B2 (also detected in our basal axonal nascent proteome; Table S3) supports axon maintenance and that the excessive axonal translation of the Activating transcription factor 4(Atf4) spreads Alzheimer’s disease pathology across the brain (Yoon et al., 2012, Baleriola et al., 2014), we investigated whether any of the axonal NSPs are linked to neurological disorders. KEGG disease analysis revealed more than 60 axonal proteins basally translated or translationally regulated in response to cue stimulation to be associated with 43 different neurological dysfunctions (Table 1). These include proteins such as Amyloid beta precursor protein (App) and Mapt, whose aggregates are associated with neurodegenerative disorders. Several proteins are related to amyotrophic lateral sclerosis (ALS), in line with recent evidence suggesting that defective axonal mRNA translation may underlie ALS progression (Murakami et al., 2015). NSPs causative of Charcot-Marie-Tooth disease were also identified in strong accord with the axon growth, pathfinding, and branching defects characterizing this pathology at early developmental stages (Ponomareva et al., 2016). These findings strengthen the idea that mislocalized mRNAs, or impaired axonal translation, constitute a possible etiology to neurological diseases.

**Table 1:** Axonal NSPs Associated with Neurological Disorders

Neurological Disease	Constitutive NSPs	Netrin-1-regulated NSPs	Sema3A-regulated NSPs	BDNF-regulated NSPs
Amyotrophic lateral sclerosis	Fus, Mapt, Hnrnpa1, Hnrnpa2b1, Prph, Vcp	Fus, Vcp, Prph, Tuba4a, Hnrnpa1, Hnrnpa2b1	Fus, Sod1, Prph, Pfn1, Tuba4a	Fus, Sod1, Prph, Vcp, Pfn1, Tuba4a, Hnrnpa1, Hnrnpa2b1
Charcot-Marie-Tooth disease	Rab7a, Aars, Gars, Vcp	Rab7a, Vcp	–	Rab7a, Aars, Vcp
Syndromic X-linked mental retardation	Syn1, L1cam, Syp	Syn1, L1cam, Syp	Syp, L1cam	L1cam, Syp, Atp6ap2
Lissencephaly	Pafah1B1, Ywhae, Reln	Pafah1B1, Ywhae	Pafah1B1, Ywhae	Pafah1B1, Ywhae, Reln
Complex cortical dysplasia with other brain malformations	Tubb, Tubb2a, Tubb3	Tubb, Tubb2a, Tubb3	Tubb, Tubb2a	Tubb2a
Primary dystonia	Atp1a3, Slc2a1	Atp1a3, Slc2a1	Atp1a3, Slc2a1	Slc2a1
Early infantile epileptic encephalopathy	Stxbp1, Gnao1, Aars	Stxbp1, Mdh2	Gnao1, Mdh2	Stxbp1, Aars
Polymicrogyria	Col18a1, Tubb2b	Col18a1, Tubb2b	Col18a1, Tubb2b	Tubb2b, Col18a1
Alzheimer’s disease	App, Mapt	App	App	App
Familial amyloidosis	Gsn, Apoa1	Gsn, Apoa1	Gsn, Apoa1	Apoa1
Adult-onset autosomal dominant leukodystrophy	Lmnb1	Lmnb1	Lmnb1	Lmnb1
Juvenile-onset dystonia	Actb	Actb	Actb	Actb
Non-syndromic X-linked mental retardation	Gdi1	Gdi1	–	–
Pierson syndrome	Lamb2, Hspd1	Lamb2	Lamb2	–
Hereditary spastic paraplegia	L1cam	L1cam, Hspd1	L1cam	L1cam, Hspd1
Progressive myoclonic epilepsy	Ctsd	Ctsd	Ctsd	Ctsd
GLUT1 deficiency syndrome	Slc2a1	Slc2a1	–	Slc2a1
Autosomal dominant mental retardation	Ctnnb1	–	–	Ctnnb1
Congenital systemic glutamine deficiency	Glul	Glul	–	Glul
Essential tremor	Fus	Fus	Fus	Fus
L1 syndrome	L1cam	L1cam	L1cam	L1cam
D-2-hydroxyglutaric aciduria	Idh2	Idh2	–	–
Retinoblastoma	Cdh11	Cdh11	–	Cdh11
Medulloblastoma	Ctnnb1	–	–	Ctnnb1
Congenital hydrocephalus	L1cam	L1cam	L1cam	L1cam
Parkinson’s disease	Mapt	–	Park7	Vps35
Frontotemporal lobar degeneration	Mapt, Vcp	Vcp	–	Vcp
Neuronal ceroid lipofuscinosis	Ctsd	Ctsd	Ctsd	Ctsd
Propionic acidemia	Pcca	–	–	Pcca
Hereditary sensory and autonomic neuropathy	Rab7a	Rab7a, Cct5	–	Rab7a
Tubb3 syndromes	Tubb3	Tubb3	–	–
Spinocerebellar ataxia	Eef2	Eef2	–	–
Limb-girdle muscular dystrophy	Dag1	Dag1	–	Dag1
Pyridoxine-dependent epilepsy	Aldh7a1	–	–	–
Ataxia telangiectasia	Pcna	–	Pcna	Pcna
Congenital mirror movements	DCC	–	–	–
Familial encephalopathy with neuroserpin inclusion bodies	Serpin I1	–	–	–
Distal hereditary motor neuropathies	Gars	–	–	–
Progressive supranuclear palsy	Mapt	–	–	–
Nemaline myopathy	Tpm3	–	–	–
AICA-ribosiduria	Atic	–	–	Atic
Krabbe disease	–	Psap	–	Psap
Leigh syndrome	–	–	Ndufs3	Pdha1

Neurological disorders were selected from the “KEGG disease” output.

## Discussion

We established pSILAC-SP3 to uncover the nascent proteome from a subcellular compartment (the axons) of growing neurons on a rapid timescale. This approach provides a direct readout of the newly synthesized proteome and can reveal information about both the magnitude and the dynamics of *de novo* protein regulation. The sensitivity of pSILAC-SP3 is sufficient to identify hundreds of NSP changes in the axonal compartment within minutes, although it is biased toward the most abundant proteins, as in any proteomics approach. It thereby contrasts with ribosome profiling, which is more sensitive due to mRNA amplification but provides a snapshot of ribosome-bound mRNAs that can be confounded by non-translated mRNAs on stalled ribosomes. The potential underestimation of the total extent of NSP changes occurring in axons as determined by pSILAC-SP3 may be further improved by increasing the length of the SILAC pulse (Eichelbaum and Krijgsveld, 2014), but this comes at the expense of temporal resolution.

Our findings revealed that protein synthesis occurs basally on a wide scale, suggesting that local translation supports proteostasis in developing axons. This agrees with the finding that proteins translated in growth cones undergo a higher degree of turnover than the more stable cell-body-derived proteins (Deglincerti et al., 2015). Ribosomal proteins represented the most-enriched category of basally translated proteins, possibly playing extra-ribosomal functions, as supported by our RpSA/67lr findings. A further possibility is that locally translated ribosomal proteins are exchanged on pre-assembled ribosomes to preserve the homeostasis of axonal ribosomes (Pulk et al., 2010, Lastick and McConkey, 1976). The basal axonal proteome also comprises several proteins belonging to metabolic pathways, including glycolysis. One such example, the glyceraldehyde-3-phosphate dehydrogenase, has recently been shown to constitute the minimal energy machinery for fast axonal transport of vesicles and to generate “glycolitic metabolons” for the support of the synaptic function (Hinckelmann et al., 2016, Jang et al., 2016). Similarly, local translation of NSPs involved in amino acid biosynthesis and mitochondrial functions may be required to replenish proteins undergoing high turnover to maintain axonal metabolism, in line with studies showing that acute inhibition of axonal protein synthesis impairs axonal mitochondria and viability in rat sympathetic neurons (Gale et al., 2018). Together, these data indicate that the developing axon, as a soma-independent subcellular compartment, helps to sustain its own biosynthetic processes and energy supply locally.

Netrin-1, Sema3A, and BDNF differentially affect axonal synthesis of multiple proteins in a cue-specific and temporally dynamic way. Interestingly, we found that the synthesis of proteasomal subunits α and β type is differentially regulated in response to different cues, raising the possibility that the degradation machinery may undergo cue-specific remodeling, leading to the formation of specialized non-canonical proteasomes with substrate selectivity. Indeed, specialized proteasomes can be generated by preferentially altering the composition of the α- (e.g., Psma4 and Psma7) and β-rings (e.g., Psmb1 and Psmb2, all detected among our cue-induced NSP changes; Padmanabhan et al., 2016, Tanaka, 2013). Similarly, some ribosomal proteins are differentially up- or downregulated in response to distinct cues, raising the possibility that extrinsic cues may also remodel the composition of pre-existing ribosomes in axons, thus tuning the translation efficiency of specific transcripts (Kondrashov et al., 2011).

We also found histones and methyltransferases to be regulated in a cue-specific manner in axons. Some histone proteins have been previously identified in the extra-nuclear compartment and histone H1 has been detected in cerebellar neurites and suggested to play a role in neurite outgrowth (Mishra et al., 2010). It is interesting to speculate that axonally synthesized histone proteins may bear distinct marks encoded by cue- or compartment-specific post-translational modifications, carrying this information back to the nucleus to modulate gene expression (Moretti et al., 2015). Alternatively, the “nuclear” proteins detected may play a novel extra-nuclear function in the axon, similar to what has been shown for LaminB2 (Yoon et al., 2012).

A question our results raise is why does cue-specific nascent proteome remodeling occur even though the cues investigated yield the same type of response (repulsive) *in vitro*? This is likely due to the activation of different receptors and downstream signaling pathways. One possibility is that different NSP changes may participate in eliciting the same chemotropic response, in line with evidence that Slit and Sema3A can induce growth cone repulsion via different mechanisms (McConnell et al., 2016). Furthermore, the *in vitro* chemotropic assay classifies cues simply as repulsive versus attractive, while cues in the developing visual pathway control a variety of protein synthesis-dependent processes, such as axonal outgrowth, branching, and synaptogenesis.

We also detected NSPs that undergo the same translational regulation in response to the three different repulsive cues, suggesting the possibility that they may be involved in common chemotropic response mechanisms. For example, the cell adhesion molecule L1cam-a, which is upregulated in response to all three repulsive cues, is required for topographic mapping of retinal axons (Demyanenko and Maness, 2003). Several tubulin isotypes also undergo common proteomic changes in response to repulsive cues but different to each other, thus supporting the “tubulin code hypothesis” proposing that tubulin isotypes possess distinctive functional properties (Tischfield and Engle, 2010). Our results also corroborate and extend the “differential translation model” for growth cone turning (Lin and Holt, 2007) as β-actin decreases on the near side of a repulsive source, leading to filopodial loss, whereas RpSA/67lr is upregulated on the far side, leading to increased adhesion and filopodial accumulation, thus helping the growth cone to turn away (Figure 6G). Indeed, recent work has shown that RpSA/67lr is crucial for directional steering at critical choice points in the retinotectal pathway (Atkinson-Leadbeater et al., 2016). Collectively, these data suggest that guidance molecules rapidly induce a coordinated set of NSP changes precisely localized across the growth cone to evoke the steering response.

Finally, we found that switching the effect of these cues from repulsion to attraction induces distinct NSP changes and differentially regulates NSPs shared between repulsion and attraction. Up to 73% of these common subsets, including, for example, NSPs belonging to the “cell adhesion,” “proteasome core complex,” and “cytoskeleton” functional clusters, undergo opposite regulation between repulsion and attraction. In the limited cytoplasmic volume of the growth cone compartment, the number of molecules must be tightly controlled in order to avoid molecular crowding; therefore, regulating a common subset of basally translated mRNAs would arguably be more efficient than controlling a completely different subset of NSPs. Moreover, the opposite regulation of the local proteome is highly reminiscent of a phenomenon uncovered in rat hippocampal cultures in which homeostatic up- and downscaling elicits opposite regulation of 46% NSPs over a 24 hr labeling period (Schanzenbächer et al., 2016). Thus, this may be a conserved biological strategy to achieve a switch between opposite responses, especially in subcellular compartments. Yet, we also observed many NSPs with the same regulation between repulsion and attraction, suggesting opposite spatial distribution within the growth cone to help the turning, or a role independent from the chemotropic response, possibly underlying branching or synaptogenesis.

In this study, we characterized the basal and rapidly cue-induced axonal nascent proteome, providing insight into the functional landscape of axon guidance and maintenance. Our work sets the stage for new studies exploring the cue-specific translational control mechanisms and the biological function of the locally synthesized proteins. Furthermore, several of the NSPs uncovered are known to be involved in neurological disorders and suggest novel possible links between defective local protein synthesis, impaired axon maintenance/wiring, and disease. Finally, guidance cues play a role also in the adult nervous system following axonal injury to inhibit (e.g., Sema3A) or promote (e.g., BDNF) axon survival and regeneration, which involve rapid local translation and degradation (Gumy et al., 2010). Therefore, our technical approach and datasets provide a framework for studies of the nascent proteome on a rapid timescale from limited amounts of material and a resource to understand neural circuit assembly and clinical pathologies.

## STAR★Methods

### Key Resources Table

**Table:** 

REAGENT or RESOURCE	SOURCE	IDENTIFIER
**Antibodies**		

Anti-β-actin	Abcam	Cat#ab6277; RRID: AB_305394
Anti-α-tubulin	Sigma	Cat#T6074; RRID: AB_477582
Anti-RpSA	Proteintech	Cat#14533-1-AP; RRID: AB_2182528
Anti-F-actin Phalloidin Alexa Fluor 488	Molecular Probes/Invitrogen	Cat#A12379; RRID: AB_2315147
Anti-puromycin	Millipore	Cat#MABE343; RRID: AB_2566826
Anti-γ-actin	Chemicon	Cat#AB3265; RRID: AB_11214345
Anti-Brn3a	Santa Cruz	Cat#sc6026; RRID: AB_673441
Anti-RpL24	Proteintech	Cat#17082-1-AP; RRID: AB_2181728
Anti-Rps23	Origene	Cat#TA314496; RRID: AB_2728701
Anti-Pdi	Sigma	Cat#P7496; RRID: AB_261952
Anti-Vamp2	Proteintech	Cat#10135-1-AP; RRID: AB_2256918
Anti-H4	Abcam	Cat#ab10158; RRID: AB_296888
Anti-H2b	Abcam	Cat#ab1790; RRID: AB_302612
Anti-Fus	Abcam	Cat#ab70381; RRID: AB_1271242
Anti-Sncg	Covance	N/A (customized)
Anti-Neurofilament-associated 3A10	Developmental Studies Hybridoma Bank	Cat#3A10; RRID: AB_531874
Anti-Glur1	Abcam	Cat#ab183797; RRID: AB_2728702
IgG isotype control	Abcam	Cat#ab27478; RRID: AB_2616600

**Chemicals, Peptides, and Recombinant Proteins**		

Netrin-1	R&D Systems	Cat#1109-N1
Sema3A	R&D Systems	Cat#1250-S3
BDNF	Sigma	Cat#B3795
Tautomycin	Calbiochem	Cat#580551
Sp-cAMP	Sigma	Cat#A166
8-Br-cGMP	Sigma	Cat#B1318
Poly-L-lysine	Sigma	Cat#P1274
Laminin	Sigma	Cat#L2020
Leibovitz L-15 medium-Lys-Arg	GIBCO Life Technologies	N/A (customized)
Stable isotope-coded amino acids Lys4	Silantes GmbH	Cat#211103913
Stable isotope-coded amino acids Lys8	Silantes GmbH	Cat#211603902
Stable isotope-coded amino acids Arg6	Silantes GmbH	Cat#201203902
Stable isotope-coded amino acids Arg10	Silantes GmbH	Cat#201603902
Puromycin	Sigma	Cat#P8833
Sera-Mag Speed Beads A	GE Healthcare	Cat#24152105050250
Sera-Mag Speed Beads B	GE Healthcare	Cat#44152105050250
Trypsin/LysC	Promega	Cat#V5071

**Critical Commercial Assays**		

RNAqueous-Micro Total RNA Isolation Kit	Invitrogen	Cat#AM1931
OneStep RT-PCR kit	QIAGEN	Cat#210210
Duolink *in situ* PLA kit	Sigma	Cat#DUO92014

**Deposited Data**		

Proteomics data	This paper	PRIDE: PXD005469

**Experimental Models: Organisms/Strains**		

*X. laevis*	Nasco	https://www.enasco.com/p/LM00535MX/

**Oligonucleotides**		

β-actin: *for* 5′ CCTGTGCAGGAAGATCACAT 3′, *rev* 5′ TGTTAAAGAGAATGAGCCCC 3′	Sigma	N/A
ɣ-actin: *for* 5′ GCGGATCTGACAGCTACTG 3′, *rev* 5′ ATCCATACAGAATGTTTTGGG 3′	Sigma	N/A
Brn3: *for* 5′ TGAGCGATTCAAGCAGAGGAGG 3′, *rev* 5′ TGCGACAGGGTGAGGGATTCAAAC 3′	Sigma	N/A
Tau: *for* 5′ TCTGCCAAGAGCCGCCTTCA 3′, *rev* 5′ GGACTGCACGTTGCCCAAATCC 3′	Sigma	N/A
Map2: *for* 5′ CGATCATCCTTGCCAAGACCTTCCTC 3′, *rev* 5′ GCGACCTGGAGATTGGGTGATGATTT 3′	Sigma	N/A
Glur1: *for* 5′ GGGATTGGCCATGCTTGTTG 3′, *rev* 5′ GCCATTCCTGCACTGTGGCTCA 3′	Sigma	N/A
Icam5: *for* 5′ TCCACCTGCAGCATTCCAGTCC 3′, *rev* 5′ TGGGGAGGCCTTCTGCATCA 3′	Sigma	N/A

**Software and Algorithms**		

Volocity	v.6.3.1	RRID: SCR_002668
GraphPad PRISM	v.5.0c	RRID: SCR_002798
ImageJ	v.149	RRID: SCR_003070
DAVID	v.6.8	RRID: SCR_001881
KEGG	N/A	RRID: SCR_012773
String	v.10.5	RRID: SCR_005223
Maxquant	v.1.4.1.2	RRID: SCR_014485
Perseus	v.1.5.1.6 or 1.5.5.3	RRID: SCR_015753

### Contact For Reagent and Resource Sharing

Further information and requests for resources and reagents should be directed to and will be fulfilled by the lead contact Christine E. Holt (ceh33@cam.ac.uk).

### Experimental Model and Subject Details

#### *Xenopus laevis* Embryos Maintenance

*Xenopus laevis* embryos of either sex were obtained by *in vitro* fertilization as previously described (Campbell and Holt, 2001), raised in 0.1x modified Barth’s saline (MBS; 0.88 mM NaCl, 0.01 mM KCl, 0.024 mM NaHCO_3_, 0.1 mM HEPES, 8.2 μM MgSO_4_, 3.3 μM Ca(NO_3_)_2_, 4.1 μM CaCl_2_) at 14–22°C and staged according to Nieuwkoop and Faber (1994). This research has been regulated under the Animals (Scientific Procedures) Act 1986 Amendment Regulations 2012 following ethical review by the University of Cambridge Animal Welfare and Ethical Review Body (AWERB).

### Method Details

#### Retinal Explant Cultures and Axotomy Assay on Transwell Filter

Whole retinas of anesthetized embryos stage 33/34-35/36 were dissected and cultured at 20°C for 24 hr in 60% L15 minimal medium (Invitrogen), 1x Penicillin Streptomycin Fungizone on glass bottom dishes (MatTek) or on the top compartment of 6-well hanging inserts with 1 μm membrane pores (Falcon), coated on both sides of the membrane with poly-L-lysine (10 μg/ml) and only on the bottom side with laminin (10 μg/ml).

For the pSILAC experiment 100 eye explants were cultured per condition. Prior to stimulation, retinal explants were removed, scraped, and washed off 7 times from the top compartment of the filter, leaving the somaless axons (∼2 μg protein typical yield) at the bottom. Each cue was added together with stable isotope-coded amino acids to the somaless axons for the time indicated. After stimulation the membrane was cut away, rinsed with ice cold PBS and lysed for protein extraction.

#### Pharmacological Treatments

Stimulations were carried out using the following concentrations: Netrin-1 (600 ng/ml), Sema3A (150 ng/ml), BDNF (200 ng/ml), Tautomycin (4 nM), Sp-cAMP (20 μM), 8-Br-cGMP (100 μM).

#### Pulsed Stable Isotope Labeling by Amino Acids in Cell Culture

All experiments were performed in at least three independent biological replicates. Retinal explants were cultured in SILAC *light* medium (Lys0, Arg0) for 24 hr and incubated in depletion medium (-Lys, -Arg) for 60 min prior pulse labeling. Subsequently, cell bodies were removed and somaless axons were incubated for the desired time (5, 15 or 30 min) with *medium* (M) (Lys4, Arg6) or *heavy* (H) isotope-coded amino acids (Lys8, Arg10). At each time point corresponding samples were lysed, immediately pooled, and processed by SP3.

#### Single-Pot Solid-Phase-Enhanced Sample Preparation

Axons were harvested by addition of lysis buffer (1% SDC, 0.1% SDS, 100 mM TrisHCl ph 8.5, 10 mM DTT, 1x protease inhibitor EDTA free). Samples were supplemented with 25 units Benzonase nuclease (Merck), and lysed in a Bioruptor (Diagenode) for 5 min (cycle 30/30, 4°C). Alkylation was performed by addition of 30 mM Chloroacetamide followed by incubation in the dark for 30 min. Protein clean-up, digestion, and peptide clean-up were performed using a modified version of the recently developed ultrasensitive sample preparation protocol SP3 (Hughes et al., 2014). In brief, 5 μL of beads (1:1 mixture of hydrophilic and hydrophobic SeraMag Carboxylate-Modified beads, GE Life Sciences) were added to each sample. Acidified acetonitrile was added to achieve a final fraction of organic solvent of 50%. Beads were incubated for 10 min to allow complete binding of proteins to the beads. Protein clean-up was performed by subsequent wash with 70% Ethanol and once with Acetonitrile. For digestion, 0.1 μg sequencing grade Trypsin/LysC (Promega) was added and digestion was performed at 37°C for 16 hr. Peptides were eluted with 9 μL 5% DMSO. 1 μL 10% formic acid was added and samples were stored at −20°C prior MS analysis.

#### Mass Spectrometry

Samples were analyzed on Orbitrap Velos Pro, Q-Exactive, or Orbitrap Fusion mass spectrometers (all Thermo Scientific) using default settings. Mass spectrometers were coupled to UPLC systems (Waters nanoAcquity UPLC or Thermo EASY LC 1200). Peptides were loaded onto trap columns (Waters nanoAcquity Symmetry C_18_, 5 μm, 180 μm × 20 mm [for Waters nanoAcquity UPLC] or Acclaim PepMap100 C18 Nano-Trap 2cm × 100 μm × 5 μm [for EASY nLC 1200]) with Buffer A (0.1% formic acid in water) and separated over 25 or 50 cm analytical columns (Acclaim PepMap RSLC, 75 μm × 2 μm) using 90, 145, or 240 min linear gradients from 3%–40% Buffer B (0.1% formic acid in Acetonitrile). Peptides were introduced into the mass spectrometer using a Pico-Tip Emitter (360 μm outer diameter × 20 μm inner diameter, 10 μm tip, New Objective). MS2 Fragmentation was set to HCD (Q-Exactive and Fusion) or CID (Orbitrap Velos Pro), and MSMS scans were acquired in the ion trap (Orbitrap Velos Pro and Fusion) or Orbitrap (Q-Exactive).

#### Proteomics Data Processing

Raw data were processed with Maxquant (version 1.4.1.2) (Cox and Mann, 2008) using default settings. MSMS spectra were searched against the *Xenopus laevis* Uniprot database (v20140925) concatenated to a database containing protein sequences of common contaminants. Enzyme specificity was set to trypsin/P, allowing a maximum of two missed cleavages. Cysteine carbamidomethylation was set as fixed modification, and methionine oxidation and protein N-terminal acetylation were used as variable modifications. The minimal peptide length allowed was set to six amino acids. The mass tolerances for peptide identification were set to 20 ppm for the first search, and 4.5 ppm for the main search. Global false discovery rates for peptide and protein identification were set to 1%. The match-between-runs and re-quantify options were enabled.

#### Reverse Transcription Polymerase Chain Reaction

RNA was extracted from using RNAqueous-Micro Total RNA Isolation Kit. Primers were designed using *Primer3Plus* software. The annealing temperature used was 58°C for β-actin and Brn3, 65°C for ɣ-actin, 64°C for Map2, GluR1 and Icam5, and 63°C for Mapt.

#### Immunocytochemistry

Retinal cultures were fixed by paraformaldehyde except for anti-β-actin (AC-15 FITC) where methanol fixation was carried out.

#### Immunohistochemistry

Embryos were euthanized and fixed overnight with 4% formaldehyde. The embryos were cryoprotected by infiltration with 30% sucrose for 1 hr, embedded in O.C.T. (Scigen) and frozen. 14 μm serial cryosections were cut and immunostained.

#### Puromycilation of NSPs and Proximity Ligation Assay

Retinal cultures were incubated with puromycin (2 ng/μl) for the condition and time (up to 15 min) of interest, fixed, and incubated with anti-puromycin antibody and the antibody against the protein of interest. Subsequently, Proximity Ligation Assay (PLA) was carried out using species-specific probes (tom Dieck et al., 2015).

#### Growth Cone Gradient Assay

Retinal explants from embryos stage 35/36 were cultured for 14-18 hr on coverslips coated with poly-L-lysine (10 μg/ml) and laminin (10 μg/ml). Sema3A (9 μg/ml) or control polarized gradients were established by pulsatile ejection as described previously (Campbell et al., 2001) for 7 min placing the micropipette at 70 μm distance and at an angle of 90° relative to the growth cone and to the initial axon shaft (Leung et al., 2006). Subsequently samples were immediately fixed and stained for β-actin and RpSA.

### Quantification and Statistical Analysis

#### Statistics

Data were analyzed with PRISM 5 (GraphPad). Data are presented as mean and error bars represent SEM. All experiments were performed in at least three independent biological replicates. Details of statistical tests used and p values are presented in the figure legends.

^∗^p ≤ 0.05, ^∗∗^p ≤ 0.01, ^∗∗∗^p ≤ 0.001, ^∗∗∗∗^p ≤ 0.0001, ns: non-significant.

#### Quantification of Immunofluorescence

For the quantification of fluorescence intensity, isolated growth cones were selected randomly with phase optics. Pixel saturation was avoided and the same gain and exposure settings were used for digital capture of images for each experiment which was performed during the same day. The outline of each single growth cone was traced using the phase image to define the region of interest (ROI) and the mean pixel intensity per unit area was measured in each channel using *Volocity* software. The background fluorescence was measured in a ROI as close as possible to the growth cone selected and subtracted to the mean fluorescence value of the growth cone.

For the growth cone gradient assay IF ratio analysis, the growth cone was bisected into two areas by a line drawn through the axon shaft and the background fluorescence level was subtracted. For the center of mass analysis, measurement was calculated as the average of all pixel locations weighted as intensity by using *ImageJ* software. The center of mass of the bright field was subtracted from the center of mass of the fluorescence signal.

#### Bioinformatic Data Analysis

For protein quantification, a minimum ratio count of 2 was set, considering unique and razor peptides. The iBAQ was calculated to determine relative abundance levels of the pre-existing light-labeled proteins. Protein ratios were log_2_-transformed, and H/M ratios of NSPs were normalized to the median using the Perseus computational framework. NSP changes were filtered applying the following criteria per each treatment, unless otherwise specified in the text: (1) quantified in >50% of biological replicates, (2) SD ≤ 1, (3) average log_2_ ratio larger than 0.3 or smaller than −0.3. Subsequently NSPs that fulfilled the following criteria were subjected to further statistical analysis. To test whether the log_2_ ratio of each NSP was significantly different from zero, p values were computed by a moderated t test implemented in the R/Bioconductor package limma (Ritchie et al., 2015). p values were corrected for multiple testing by controlling the false discovery rate with the method of Benjamini-Hochberg. Hierarchical clustering of proteins was performed in Perseus on log_2_ transformed ratios, using Euclidean distances and average linkage. Enrichment of categorical annotations (Gene Ontology) was determined using DAVID. Pathway and disease analyses were carried out using KEGG. Interaction network analysis was obtained by employing String v10.5 database (Szklarczyk et al., 2017). Each node represents a NSP change and each edge shows protein-protein interaction. Proteins with related functions were clustered together based on enrichment of categorical annotations (Gene Ontology) obtained using DAVID and disconnected nodes were removed from the plot for simplicity.

### Data and Software Availability

The accession number for the mass spectrometry proteomics data reported in this paper is ProteomeXchange Consortium via PRIDE (Vizcaíno et al., 2016): PXD005469.

## Supporting Citations

The following references appear in the Supplemental Information: Batista et al., 2017, Blichenberg et al., 1999, Eng et al., 1999, Grooms et al., 2006, Gumy et al., 2011, Kastenhuber et al., 2009, Kessler and Baude, 1999, Nadal-Nicolás et al., 2009, Nicolaï et al., 2010, Preitner et al., 2014, Schwanhäusser et al., 2011, Surgucheva et al., 2008, Taylor et al., 2013.

## Supplemental Information

Document S1. Figures S1–S7 and Tables S1 and S2

Table S3. Basal and Cue-Induced Nascent Axonal Proteomes, Related to Figures 1–7

Table S4. Cue-Induced Nascent Axonal Proteomes, Related to Figure 7

Table S5. Cue-Induced NSP Changes in Common between Repulsion and Attraction, Related to Figure 7

Table S6. Sequences of All Identified Peptides, Including Scoring Metrics and Raw Intensities, and Overlap between Replicates on Peptide and Protein Level, Related to Figures 1–7

Document S2. Article plus Supplemental Information

## References

[bib1] Aguado F., Carmona M.A., Pozas E., Aguiló A., Martínez-Guijarro F.J., Alcantara S., Borrell V., Yuste R., Ibañez C.F., Soriano E. (2003). BDNF regulates spontaneous correlated activity at early developmental stages by increasing synaptogenesis and expression of the K+/Cl- co-transporter KCC2. Development.

[bib2] Atkinson-Leadbeater K., Hehr C.L., Johnston J., Bertolesi G., McFarlane S. (2016). EGCG stabilizes growth cone filopodia and impairs retinal ganglion cell axon guidance. Dev. Dyn..

[bib3] Baleriola J., Walker C.A., Jean Y.Y., Crary J.F., Troy C.M., Nagy P.L., Hengst U. (2014). Axonally synthesized ATF4 transmits a neurodegenerative signal across brain regions. Cell.

[bib4] Batista A.F.R., Martínez J.C., Hengst U. (2017). Intra-axonal synthesis of SNAP25 is required for the formation of presynaptic terminals. Cell Rep..

[bib5] Bellon A., Iyer A., Bridi S., Lee F.C.Y., Ovando-Vázquez C., Corradi E., Longhi S., Roccuzzo M., Strohbuecker S., Naik S. (2017). miR-182 regulates Slit2-mediated axon guidance by modulating the local translation of a specific mRNA. Cell Rep..

[bib6] Biederer T., Sara Y., Mozhayeva M., Atasoy D., Liu X., Kavalali E.T., Südhof T.C. (2002). SynCAM, a synaptic adhesion molecule that drives synapse assembly. Science.

[bib7] Blichenberg A., Schwanke B., Rehbein M., Garner C.C., Richter D., Kindler S. (1999). Identification of a cis-acting dendritic targeting element in MAP2 mRNAs. J. Neurosci..

[bib8] Campbell D.S., Holt C.E. (2001). Chemotropic responses of retinal growth cones mediated by rapid local protein synthesis and degradation. Neuron.

[bib9] Campbell D.S., Regan A.G., Lopez J.S., Tannahill D., Harris W.A., Holt C.E. (2001). Semaphorin 3A elicits stage-dependent collapse, turning, and branching in Xenopus retinal growth cones. J. Neurosci..

[bib10] Cheng P.L., Song A.H., Wong Y.H., Wang S., Zhang X., Poo M.M. (2011). Self-amplifying autocrine actions of BDNF in axon development. Proc. Natl. Acad. Sci. USA.

[bib11] Cohen-Cory S., Fraser S.E. (1995). Effects of brain-derived neurotrophic factor on optic axon branching and remodelling in vivo. Nature.

[bib12] Cox J., Mann M. (2008). MaxQuant enables high peptide identification rates, individualized p.p.b.-range mass accuracies and proteome-wide protein quantification. Nat. Biotechnol..

[bib13] Deglincerti A., Liu Y., Colak D., Hengst U., Xu G., Jaffrey S.R. (2015). Coupled local translation and degradation regulate growth cone collapse. Nat. Commun..

[bib14] Demyanenko G.P., Maness P.F. (2003). The L1 cell adhesion molecule is essential for topographic mapping of retinal axons. J. Neurosci..

[bib15] Dieterich D.C., Link A.J., Graumann J., Tirrell D.A., Schuman E.M. (2006). Selective identification of newly synthesized proteins in mammalian cells using bioorthogonal noncanonical amino acid tagging (BONCAT). Proc. Natl. Acad. Sci. USA.

[bib16] Eichelbaum K., Krijgsveld J. (2014). Rapid temporal dynamics of transcription, protein synthesis, and secretion during macrophage activation. Mol. Cell. Proteomics.

[bib17] Eng H., Lund K., Campenot R.B. (1999). Synthesis of beta-tubulin, actin, and other proteins in axons of sympathetic neurons in compartmented cultures. J. Neurosci..

[bib18] Firestone A.J., Weinger J.S., Maldonado M., Barlan K., Langston L.D., O’Donnell M., Gelfand V.I., Kapoor T.M., Chen J.K. (2012). Small-molecule inhibitors of the AAA+ ATPase motor cytoplasmic dynein. Nature.

[bib19] Gale J.R., Aschrafi A., Gioio A.E., Kaplan B.B. (2018). Nuclear-encoded mitochondrial mRNAs: a powerful force in axonal growth and development. Neuroscientist.

[bib20] Grooms S.Y., Noh K.M., Regis R., Bassell G.J., Bryan M.K., Carroll R.C., Zukin R.S. (2006). Activity bidirectionally regulates AMPA receptor mRNA abundance in dendrites of hippocampal neurons. J. Neurosci..

[bib21] Gumy L.F., Tan C.L., Fawcett J.W. (2010). The role of local protein synthesis and degradation in axon regeneration. Exp. Neurol..

[bib22] Gumy L.F., Yeo G.S., Tung Y.C., Zivraj K.H., Willis D., Coppola G., Lam B.Y., Twiss J.L., Holt C.E., Fawcett J.W. (2011). Transcriptome analysis of embryonic and adult sensory axons reveals changes in mRNA repertoire localization. RNA.

[bib23] Gygi S.P., Rochon Y., Franza B.R., Aebersold R. (1999). Correlation between protein and mRNA abundance in yeast. Mol. Cell. Biol..

[bib24] Harris W.A., Holt C.E., Bonhoeffer F. (1987). Retinal axons with and without their somata, growing to and arborizing in the tectum of Xenopus embryos: a time-lapse video study of single fibres in vivo. Development.

[bib25] He Y., Ren Y., Wu B., Decourt B., Lee A.C., Taylor A., Suter D.M. (2015). Src and cortactin promote lamellipodia protrusion and filopodia formation and stability in growth cones. Mol. Biol. Cell.

[bib26] Hengst U., Deglincerti A., Kim H.J., Jeon N.L., Jaffrey S.R. (2009). Axonal elongation triggered by stimulus-induced local translation of a polarity complex protein. Nat. Cell Biol..

[bib27] Hinckelmann M.V., Virlogeux A., Niehage C., Poujol C., Choquet D., Hoflack B., Zala D., Saudou F. (2016). Self-propelling vesicles define glycolysis as the minimal energy machinery for neuronal transport. Nat. Commun..

[bib28] Höpker V.H., Shewan D., Tessier-Lavigne M., Poo M., Holt C. (1999). Growth-cone attraction to netrin-1 is converted to repulsion by laminin-1. Nature.

[bib29] Huang Y., Higginson D.S., Hester L., Park M.H., Snyder S.H. (2007). Neuronal growth and survival mediated by eIF5A, a polyamine-modified translation initiation factor. Proc. Natl. Acad. Sci. USA.

[bib30] Hughes C.S., Foehr S., Garfield D.A., Furlong E.E., Steinmetz L.M., Krijgsveld J. (2014). Ultrasensitive proteome analysis using paramagnetic bead technology. Mol. Syst. Biol..

[bib31] Igarashi M., Strittmatter S.M., Vartanian T., Fishman M.C. (1993). Mediation by G proteins of signals that cause collapse of growth cones. Science.

[bib32] Jain S., Welshhans K. (2016). Netrin-1 induces local translation of down syndrome cell adhesion molecule in axonal growth cones. Dev. Neurobiol..

[bib33] Jamieson K.V., Wu J., Hubbard S.R., Meruelo D. (2008). Crystal structure of the human laminin receptor precursor. J. Biol. Chem..

[bib34] Jang S., Nelson J.C., Bend E.G., Rodríguez-Laureano L., Tueros F.G., Cartagenova L., Underwood K., Jorgensen E.M., Colón-Ramos D.A. (2016). Glycolytic enzymes localize to synapses under energy stress to support synaptic function. Neuron.

[bib35] Kastenhuber E., Kern U., Bonkowsky J.L., Chien C.B., Driever W., Schweitzer J. (2009). Netrin-DCC, Robo-Slit, and heparan sulfate proteoglycans coordinate lateral positioning of longitudinal dopaminergic diencephalospinal axons. J. Neurosci..

[bib36] Kessler J.P., Baude A. (1999). Distribution of AMPA receptor subunits GluR1-4 in the dorsal vagal complex of the rat: a light and electron microscope immunocytochemical study. Synapse.

[bib37] Kim H.W., Ha S.H., Lee M.N., Huston E., Kim D.H., Jang S.K., Suh P.G., Houslay M.D., Ryu S.H. (2010). Cyclic AMP controls mTOR through regulation of the dynamic interaction between Rheb and phosphodiesterase 4D. Mol. Cell. Biol..

[bib38] Kondrashov N., Pusic A., Stumpf C.R., Shimizu K., Hsieh A.C., Ishijima J., Shiroishi T., Barna M. (2011). Ribosome-mediated specificity in Hox mRNA translation and vertebrate tissue patterning. Cell.

[bib39] Lastick S.M., McConkey E.H. (1976). Exchange and stability of HeLa ribosomal proteins in vivo. J. Biol. Chem..

[bib40] Lepelletier L., Langlois S.D., Kent C.B., Welshhans K., Morin S., Bassell G.J., Yam P.T., Charron F. (2017). Sonic Hedgehog guides axons via Zipcode Binding Protein 1-mediated local translation. J. Neurosci..

[bib41] Leung K.M., van Horck F.P., Lin A.C., Allison R., Standart N., Holt C.E. (2006). Asymmetrical beta-actin mRNA translation in growth cones mediates attractive turning to netrin-1. Nat. Neurosci..

[bib42] Lin A.C., Holt C.E. (2007). Local translation and directional steering in axons. EMBO J..

[bib43] Litman P., Barg J., Rindzoonski L., Ginzburg I. (1993). Subcellular localization of tau mRNA in differentiating neuronal cell culture: implications for neuronal polarity. Neuron.

[bib44] Lyles V., Zhao Y., Martin K.C. (2006). Synapse formation and mRNA localization in cultured Aplysia neurons. Neuron.

[bib45] Manitt C., Nikolakopoulou A.M., Almario D.R., Nguyen S.A., Cohen-Cory S. (2009). Netrin participates in the development of retinotectal synaptic connectivity by modulating axon arborization and synapse formation in the developing brain. J. Neurosci..

[bib46] McConnell R.E., Edward van Veen J., Vidaki M., Kwiatkowski A.V., Meyer A.S., Gertler F.B. (2016). A requirement for filopodia extension toward Slit during Robo-mediated axon repulsion. J. Cell Biol..

[bib47] Mishra B., von der Ohe M., Schulze C., Bian S., Makhina T., Loers G., Kleene R., Schachner M. (2010). Functional role of the interaction between polysialic acid and extracellular histone H1. J. Neurosci..

[bib48] Moretti F., Rolando C., Winker M., Ivanek R., Rodriguez J., Von Kriegsheim A., Taylor V., Bustin M., Pertz O. (2015). Growth cone localization of the mRNA encoding the chromatin regulator HMGN5 modulates neurite outgrowth. Mol. Cell. Biol..

[bib49] Murakami T., Qamar S., Lin J.Q., Schierle G.S., Rees E., Miyashita A., Costa A.R., Dodd R.B., Chan F.T., Michel C.H. (2015). ALS/FTD mutation-induced phase transition of FUS liquid droplets and reversible hydrogels into irreversible hydrogels impairs RNP granule function. Neuron.

[bib50] Nadal-Nicolás F.M., Jiménez-López M., Sobrado-Calvo P., Nieto-López L., Cánovas-Martínez I., Salinas-Navarro M., Vidal-Sanz M., Agudo M. (2009). Brn3a as a marker of retinal ganglion cells: qualitative and quantitative time course studies in naive and optic nerve-injured retinas. Invest. Ophthalmol. Vis. Sci..

[bib51] Nakaya N., Sultana A., Lee H.S., Tomarev S.I. (2012). Olfactomedin 1 interacts with the Nogo A receptor complex to regulate axon growth. J. Biol. Chem..

[bib52] Nicolaï L.J., Ramaekers A., Raemaekers T., Drozdzecki A., Mauss A.S., Yan J., Landgraf M., Annaert W., Hassan B.A. (2010). Genetically encoded dendritic marker sheds light on neuronal connectivity in Drosophila. Proc. Natl. Acad. Sci. USA.

[bib53] Nieuwkoop, P.D., and Faber, J. (1994). Normal table of Xenopus laevis (Daudin): A Systematical and Chronological Survey of the Development from the Fertilized Egg till the End of Metamorphosis (Garland).

[bib54] Padmanabhan A., Vuong S.A., Hochstrasser M. (2016). Assembly of an evolutionarily conserved alternative proteasome isoform in human cells. Cell Rep..

[bib55] Piper M., Anderson R., Dwivedy A., Weinl C., van Horck F., Leung K.M., Cogill E., Holt C. (2006). Signaling mechanisms underlying Slit2-induced collapse of Xenopus retinal growth cones. Neuron.

[bib56] Ponomareva O.Y., Eliceiri K.W., Halloran M.C. (2016). Charcot-Marie-Tooth 2b associated Rab7 mutations cause axon growth and guidance defects during vertebrate sensory neuron development. Neural Dev..

[bib57] Preitner N., Quan J., Nowakowski D.W., Hancock M.L., Shi J., Tcherkezian J., Young-Pearse T.L., Flanagan J.G. (2014). APC is an RNA-binding protein, and its interactome provides a link to neural development and microtubule assembly. Cell.

[bib58] Pulk A., Liiv A., Peil L., Maiväli U., Nierhaus K., Remme J. (2010). Ribosome reactivation by replacement of damaged proteins. Mol. Microbiol..

[bib59] Rajasundaram D., Selbig J., Persson S., Klie S. (2014). Co-ordination and divergence of cell-specific transcription and translation of genes in arabidopsis root cells. Ann. Bot. (Lond.).

[bib60] Ritchie M.E., Phipson B., Wu D., Hu Y., Law C.W., Shi W., Smyth G.K. (2015). limma powers differential expression analyses for RNA-sequencing and microarray studies. Nucleic Acids Res..

[bib61] Schanzenbächer C.T., Sambandan S., Langer J.D., Schuman E.M. (2016). Nascent proteome remodeling following homeostatic scaling at hippocampal synapses. Neuron.

[bib62] Schwanhäusser B., Gossen M., Dittmar G., Selbach M. (2009). Global analysis of cellular protein translation by pulsed SILAC. Proteomics.

[bib63] Schwanhäusser B., Busse D., Li N., Dittmar G., Schuchhardt J., Wolf J., Chen W., Selbach M. (2011). Global quantification of mammalian gene expression control. Nature.

[bib64] Shen Y., Mani S., Donovan S.L., Schwob J.E., Meiri K.F. (2002). Growth-associated protein-43 is required for commissural axon guidance in the developing vertebrate nervous system. J. Neurosci..

[bib65] Shigeoka T., Jung H., Jung J., Turner-Bridger B., Ohk J., Lin J.Q., Amieux P.S., Holt C.E. (2016). Dynamic axonal translation in developing and mature visual circuits. Cell.

[bib66] Song H.J., Ming G.L., Poo M.M. (1997). cAMP-induced switching in turning direction of nerve growth cones. Nature.

[bib67] Song H., Ming G., He Z., Lehmann M., McKerracher L., Tessier-Lavigne M., Poo M. (1998). Conversion of neuronal growth cone responses from repulsion to attraction by cyclic nucleotides. Science.

[bib68] Surgucheva I., Weisman A.D., Goldberg J.L., Shnyra A., Surguchov A. (2008). Gamma-synuclein as a marker of retinal ganglion cells. Mol. Vis..

[bib69] Szklarczyk D., Morris J.H., Cook H., Kuhn M., Wyder S., Simonovic M., Santos A., Doncheva N.T., Roth A., Bork P. (2017). The STRING database in 2017: quality-controlled protein-protein association networks, made broadly accessible. Nucleic Acids Res..

[bib70] Tachibana H., Koga K., Fujimura Y., Yamada K. (2004). A receptor for green tea polyphenol EGCG. Nat. Struct. Mol. Biol..

[bib71] Takahashi H., Craig A.M. (2013). Protein tyrosine phosphatases PTPδ, PTPσ, and LAR: presynaptic hubs for synapse organization. Trends Neurosci..

[bib72] Tanaka K. (2013). The proteasome: from basic mechanisms to emerging roles. Keio J. Med..

[bib73] Taylor A.M., Wu J., Tai H.C., Schuman E.M. (2013). Axonal translation of β-catenin regulates synaptic vesicle dynamics. J. Neurosci..

[bib74] Tischfield M.A., Engle E.C. (2010). Distinct alpha- and beta-tubulin isotypes are required for the positioning, differentiation and survival of neurons: new support for the ‘multi-tubulin’ hypothesis. Biosci. Rep..

[bib75] tom Dieck S., Kochen L., Hanus C., Heumüller M., Bartnik I., Nassim-Assir B., Merk K., Mosler T., Garg S., Bunse S. (2015). Direct visualization of newly synthesized target proteins in situ. Nat. Methods.

[bib76] Umeda D., Yano S., Yamada K., Tachibana H. (2008). Green tea polyphenol epigallocatechin-3-gallate signaling pathway through 67-kDa laminin receptor. J. Biol. Chem..

[bib77] Vidaki M., Drees F., Saxena T., Lanslots E., Taliaferro M.J., Tatarakis A., Burge C.B., Wang E.T., Gertler F.B. (2017). A requirement for Mena, an actin regulator, in local mRNA translation in developing neurons. Neuron.

[bib78] Villarin J.M., McCurdy E.P., Martínez J.C., Hengst U. (2016). Local synthesis of dynein cofactors matches retrograde transport to acutely changing demands. Nat. Commun..

[bib79] Vizcaíno J.A., Csordas A., del-Toro N., Dianes J.A., Griss J., Lavidas I., Mayer G., Perez-Riverol Y., Reisinger F., Ternent T. (2016). 2016 update of the PRIDE database and its related tools. Nucleic Acids Res..

[bib80] Wen Z., Guirland C., Ming G.L., Zheng J.Q. (2004). A CaMKII/calcineurin switch controls the direction of Ca(2+)-dependent growth cone guidance. Neuron.

[bib81] Willis D.E., Twiss J.L. (2011). Profiling axonal mRNA transport. Methods Mol. Biol..

[bib82] Wong H.H., Lin J.Q., Ströhl F., Roque C.G., Cioni J.M., Cagnetta R., Turner-Bridger B., Laine R.F., Harris W.A., Kaminski C.F., Holt C.E. (2017). RNA docking and local translation regulate site-specific axon remodeling in vivo. Neuron.

[bib83] Wu K.Y., Hengst U., Cox L.J., Macosko E.Z., Jeromin A., Urquhart E.R., Jaffrey S.R. (2005). Local translation of RhoA regulates growth cone collapse. Nature.

[bib84] Yao J., Sasaki Y., Wen Z., Bassell G.J., Zheng J.Q. (2006). An essential role for beta-actin mRNA localization and translation in Ca2+-dependent growth cone guidance. Nat. Neurosci..

[bib85] Yoon B.C., Jung H., Dwivedy A., O’Hare C.M., Zivraj K.H., Holt C.E. (2012). Local translation of extranuclear lamin B promotes axon maintenance. Cell.

[bib86] Zielinski J., Kilk K., Peritz T., Kannanayakal T., Miyashiro K.Y., Eiríksdóttir E., Jochems J., Langel U., Eberwine J. (2006). In vivo identification of ribonucleoprotein-RNA interactions. Proc. Natl. Acad. Sci. USA.

[bib87] Zivraj K.H., Tung Y.C., Piper M., Gumy L., Fawcett J.W., Yeo G.S., Holt C.E. (2010). Subcellular profiling reveals distinct and developmentally regulated repertoire of growth cone mRNAs. J. Neurosci..

[bib88] Zylbersztejn K., Petkovic M., Burgo A., Deck M., Garel S., Marcos S., Bloch-Gallego E., Nothias F., Serini G., Bagnard D. (2012). The vesicular SNARE Synaptobrevin is required for Semaphorin 3A axonal repulsion. J. Cell Biol..

