# Supplementary material for: Rapid Cue-Specific Remodeling of the Nascent Axonal Proteome
Source: Neuron. 2018 Jul 11;99(1):29–46.e4. doi: 10.1016/j.neuron.2018.06.004 (PMC6048689; doi:10.1016/j.neuron.2018.06.004)
Supplement: Document S1. Figures S1–S7 and Tables S1 and S2 [file mmc1.pdf]

Neuron, Volume *99*

Supplemental Information

Rapid Cue-Specific Remodeling of the Nascent Axonal Proteome

Roberta Cagnetta, Christian K. Frese, Toshiaki Shigeoka, Jeroen Krijgsveld, and Christine E. Holt

Supplemental Figure 1

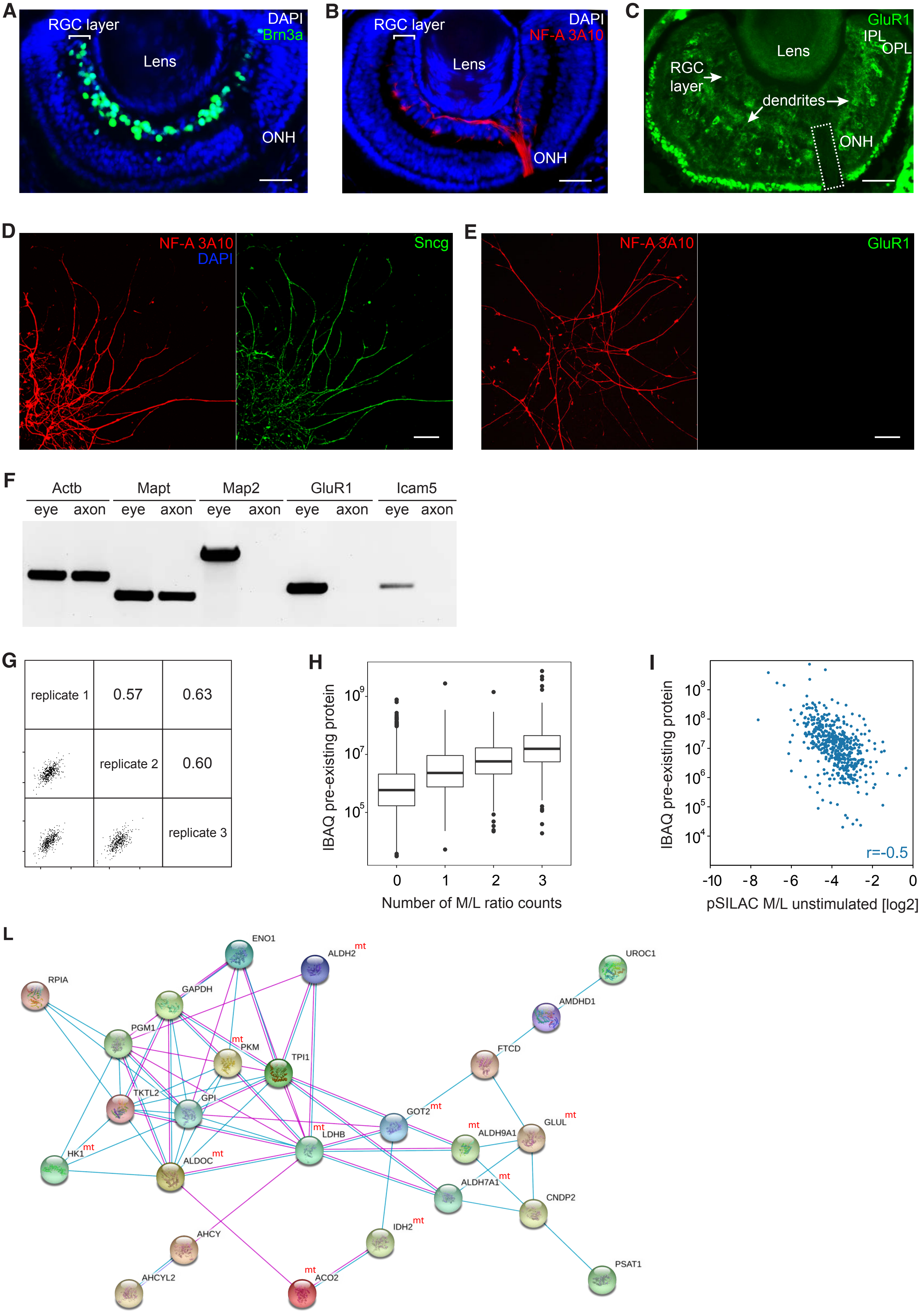

Figure S1. Analysis of the basal newly synthesized proteome after 5 min pSILAC – Related to Figure 1

(A) Embryo retinal section was counterstained for the nuclear marker DAPI and the RGC marker Brn3a (Nadal-Nicolas et al., 2009), thus identifying the RGC layer. **(B)** Embryo retinal section was counterstained for the nuclear marker DAPI and the axonal marker Neurofilament-associated 3A10 (NF-A 3A10; Kastenhuber et al., 2009). All the axons leaving the eye through the ONH derive from the RGC layer. **(C)** Embryo retinal section was stained for the somatodendritic marker Glutamate Receptor 1 (GluR1; Kessler and Baude, 1999). GluR1 was not detected in the ONH**.** **(D)** Retinal explants were cultured on the boyden chamber, isolated from the eye and counterstained for DAPI, the axonal marker NF-A 3A10 and the RGC marker γ-synuclein (Sncg; Surgucheva et al., 2008). Stack image throughout the transfilter shows that no DAPI was detected and all the axons were positive to Sncg. **(E)** Retinal explants were cultured on the boyden chamber, isolated from the eye and stained for the axonal marker NF-A 3A10 and the somatodendritic marker GluR1. Stack image throughout the transfilter shows that all the axons were negative to GluR1. **(F)** RT-PCR confirms the purity of the axonal compartment. The positive control Actb (Leung et al., 2006) and the axonal marker Mapt/Tau (Litman et al., 1993) were detected. The dendritic markers Map2, GluR1 and Icam5 (Blichenberg et al., 1999; Grooms et al., 2006; Nicolaï et al., 2010) were absent in the axonal sample. **(G)** Multi scatter plot illustrating reproducibility for 3 independent biological replicates of 5 min pSILAC labeling. Numbers correspond to PCC. **(H)** Number of M/L ratio counts *vs* Intensity Based Absolute Quantification (IBAQ) as measure of protein abundance of preexisting proteins (Schwanhausser et al., 2011). **(I)** $\log_2$ ratios of constitutive axonal NSPs over pre-existing proteins *vs* IBAQ of preexisting proteins (Schwanhausser et al., 2011) reveals a negative correlation. **(L)** STRING-based interactome of the basal NSPs belonging to enriched KEGG metabolic pathways. Nodes represent NSPs acting in the histidine metabolism, selenoamino acid metabolism, biosynthesis of amino acids and glycolysis/gluconeogenesis pathways; ‘mt’ indicates proteins belonging to the GO cellular composition categories ‘mitochondrion’ and ‘mitochondrial matrix’; light blue lines indicate interactions known from databases, purple lines indicate interactions experimentally determined. RGC: Retinal ganglion cell; ONH: Optic Nerve Head; IPL: Inner Plexiform Layer; OPL: Outer Plexiform Layer. Scale bar Figure S1A-C: 25 μm, S1D-E: 50 μm.

Supplemental Figure 2

A

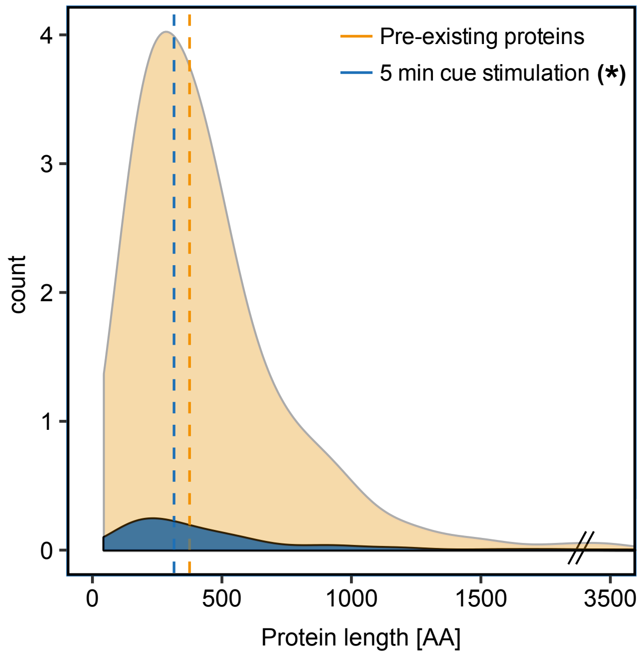

B

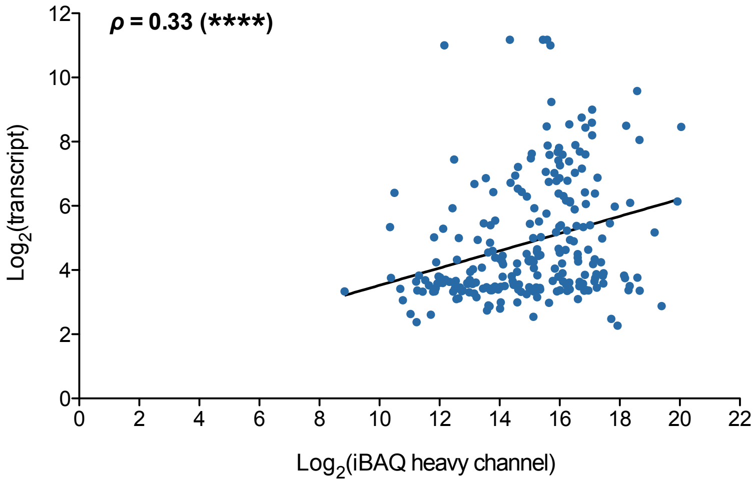

Figure S2. Analysis of the cue-induced newly synthesized proteome – Related to Figure 2

(A) Protein size distribution of the axonal proteome and of NSPs up-regulated in response to 5 min cue stimulation. The NSPs up-regulated within 5 min stimulation are shifted slightly towards the shorter size end (Kolmogorov-Smirnov test). **(B)** Analysis of the transcriptome abundance previously detected in *Xenopus* stage 32 growth cones (Zivraj et al., 2010) *versus* the cue-induced IBAQ (Schwanhausser et al., 2011) shows significant positive correlation (Spearman's rank correlation coefficient $\rho = 0.33$).

Supplemental Figure 3

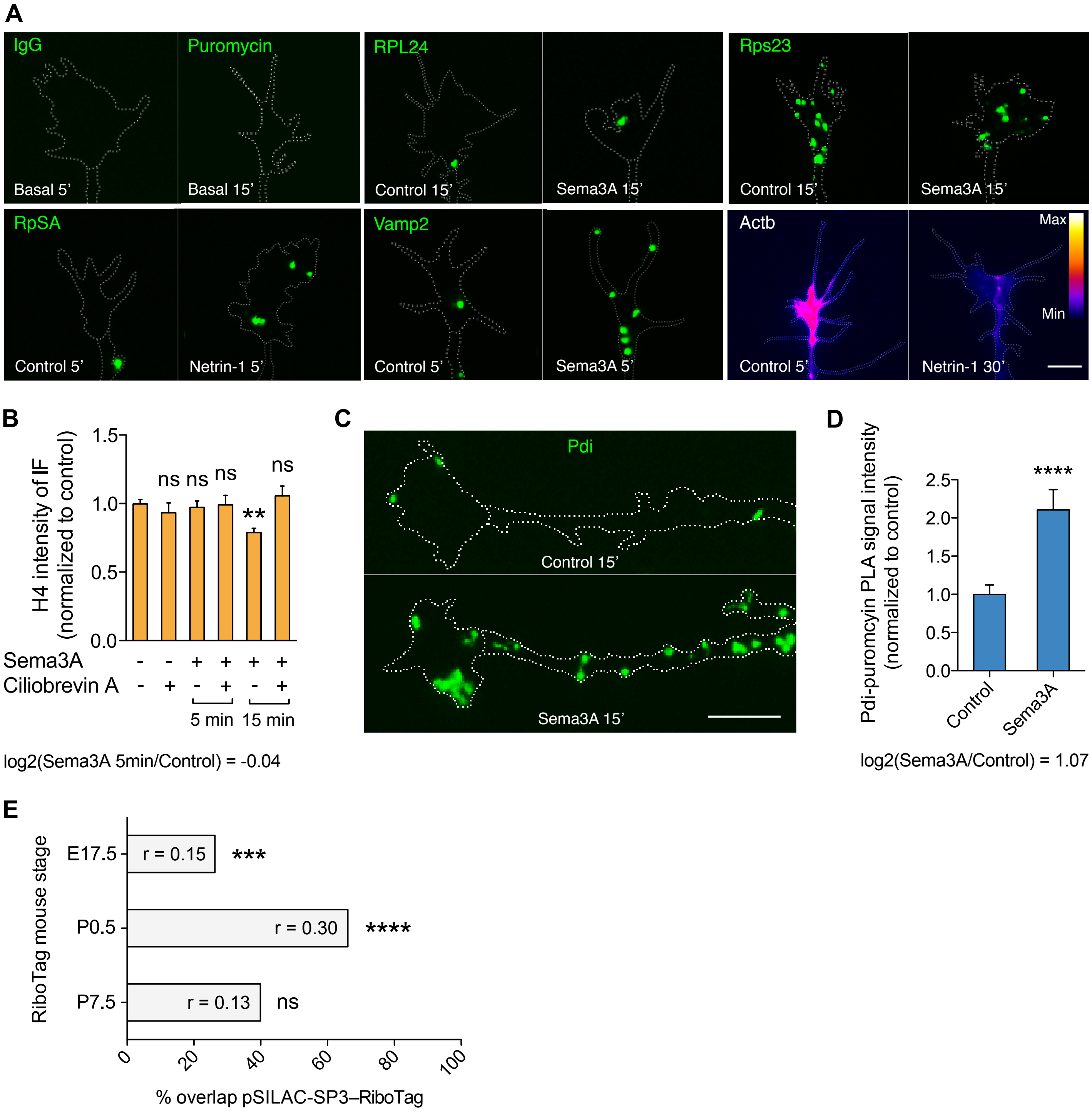

Figure S3. Validation of the pSILAC-SP3 approach – Related to Figure 3

(A) puro-PLA and IF representative images. **(B)** Growth cones were stimulated with Sema3A and co-treated with Ciliobrevin A, stained for Histone H4, and IF was measured. H4 total protein level does not change following 5 min Sema3A stimulation and decreases following 15 min Sema3A stimulation. This decrease is blocked by Ciliobrevin A (Mann-Whitney test and one-way ANOVA with Bonferroni's Multiple Comparison test). **(C-D)** Pdi puro-PLA in response to 15 min Sema3A stimulation measured along 20 μm of the axon proximal to the growth cone. Sema3A increases Pdi axonal translation. The outcome perfectly correlates with the pSILAC outcome (Table S1) and the puro-PLA measurement in growth cones (Figure 3F) (Mann-Whitney test). **(E)** Percentage of overlap and abundance correlation (r) between the pSILAC-SP3 outcome in response to cues and the mRNAs detected to be associated with ribosomes in the RiboTag at different developmental stages *in vivo* (Chi-square test). Scale bars 5 μm.

Table S1

Protein	Condition	log_2(Condition/Control)	p-value	mRNA
Actg	Basal	-		U[1,5]
Brn3a	Basal	-		U[1,4]
RpL24	Basal	-2.15		D[1]
RpL24	Sema3A 15’	NaN		D[1]
RpS23	Basal	NaN		D[1]
RpS23	Sema3A 15’	NaN		D[1]
Pdi	Sema3A 15’	1.03	0.10	D[1]
Pdi	Netrin-1 15’	-1.67	0.09	D[1]
RpSA	Sema3A 5’	1.32	0.00	D[1]
RpSA	Netrin-1 5’	0.42	0.32	D[1]
Vamp2	Sema3A 5’	0.87	0.07	U
H4	Sema3A 5’	1.13	0.06	D[2,3]
H2b	Sema3A 5’	1.09	0.02	D[3]
Fus	BDNF 30’	-0.79	0.18	D[1]
Tuba	Sema3A 15’	-1.63 (Tuba1a)	0.00	D[3]
		-1.75 (Tuba3c)	0.00	D[1]
Actb	Netrin-1 5’	-1.34	0.00	D[1]
Actb	Netrin-1 15’	-0.89	0.09	D[1]
Actb	Netrin-1 30’	-0.14	NaN	D[1]
Actb	BDNF 5’	-0.53	NaN	D[1]
Actb	Sema3A 5’	-2.27	NaN	D[1]

Table S1. pSILAC and transcriptome outcome of the proteins selected for validation – Related to Figure 3

Table shows the pSILAC-derived NSP changes of the proteins selected for validation. It has to be noted that the ratio $\log_2$('Medium'/'Light') corresponding to basal translation always exhibits negative value due to the predominance of pre-existing proteins (i.e.'Light'). Green indicates validated NSP changes, black indicates not validated NSP changes likely due to bordeline detection by pSILAC-SP3 (NaN: Not a Number). The mRNA column indicates if the corresponding transcript has been detected (D) or undetected (U) in axons by previous work: (1) Zivraj et al., 2010; (2) experimentally detected by RT-PCR; (3) Gumy et al., 2011; (4) Yoon et al., 2012; (5) Willis and Twiss, 2011.

Table S2

Protein	Condition	log_2(Condition/Control)	System	Reference	RiboTag
Tubb	Basal	-5.27	Rat sympathetic axons	Eng et al., 1999	D
Tubb2b	Basal	-4.97	Mouse dorsal root ganglion growth cones	Preitner et al., 2014	D
Pafah1b1	Basal	-3.96	Rat dorsal root ganglion axons	Villarin et al., 2016	D
Lmnb2	Basal	-2.66	Xenopus retinal growth cones	Yoon et al., 2012	U
Ctnnb1	Basal	-5.29	Rat hippocampal axons	Taylor et al., 2013	D
Snap25	Basal	-3.14	Rat hippocampal axons	Batista et al., 2017	D
Actb	BDNF 5’	-0.53	Xenopus spinal growth cones	Yao et al., 2006	D
Cofilin	Sema3A 5’	0.82 (Cfl-1a)	Xenopus retinal growth cones	Piper et al., 2006	D
Dyrk1a	BDNF 15’	NaN	Mouse cortical growth cones	Vidaki et al., 2017	D
Dscam	Netrin-1 30’	NaN	Mouse hippocampal growth cones	Jain and Welshhans, 2016	D

Table S2. Comparison of the pSILAC outcome with previous findings – Related to Figure 3

Table shows the pSILAC-derived NSP changes compared to previous findings and the RiboTag axonal translatome (Shigeoka et al., 2016), D: detected, U: undetected. It has to be noted that the ratio log_2('Medium'/'Light') corresponding to basal translation always exhibits negative value due to the predominance of pre-existing proteins (i.e.'Light'). Green indicates NSP changes in accord with previous findings, black indicates NSP changes not matching with previous findings likely due either to borderline detection by pSILAC-SP3 or to inter-species differences. NaN: Not a Number.

Supplemental Figure 4

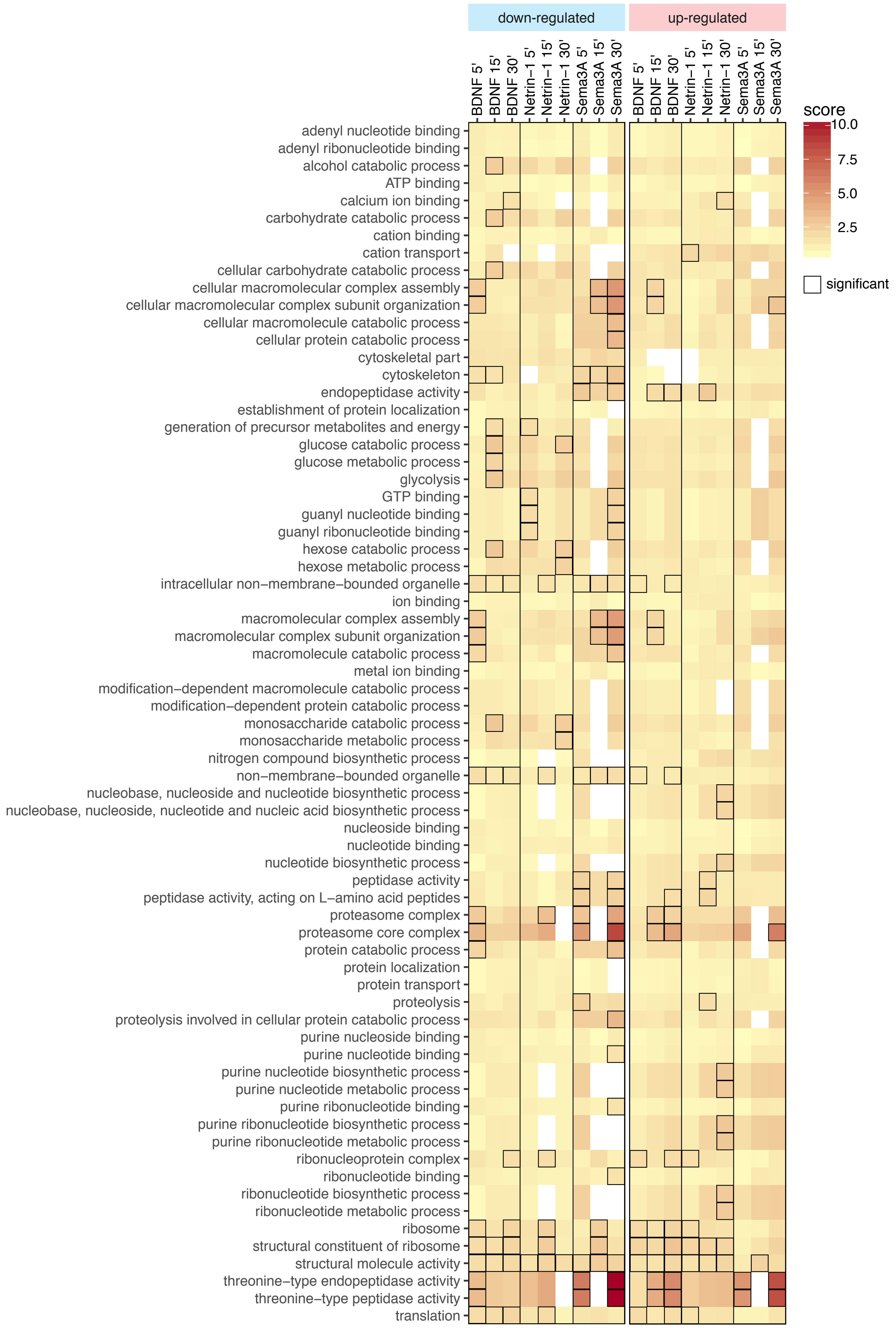

Figure S4. Functional enrichment analysis of the cue-induced newly synthesized proteomes after different stimulation times – Related to Figure 4

Enriched GO terms in the biological process, molecular function and cellular composition categories (category count > 15). Rectangles indicate significantly enriched GO terms (p-value < 0.05).

Supplemental Figure 5

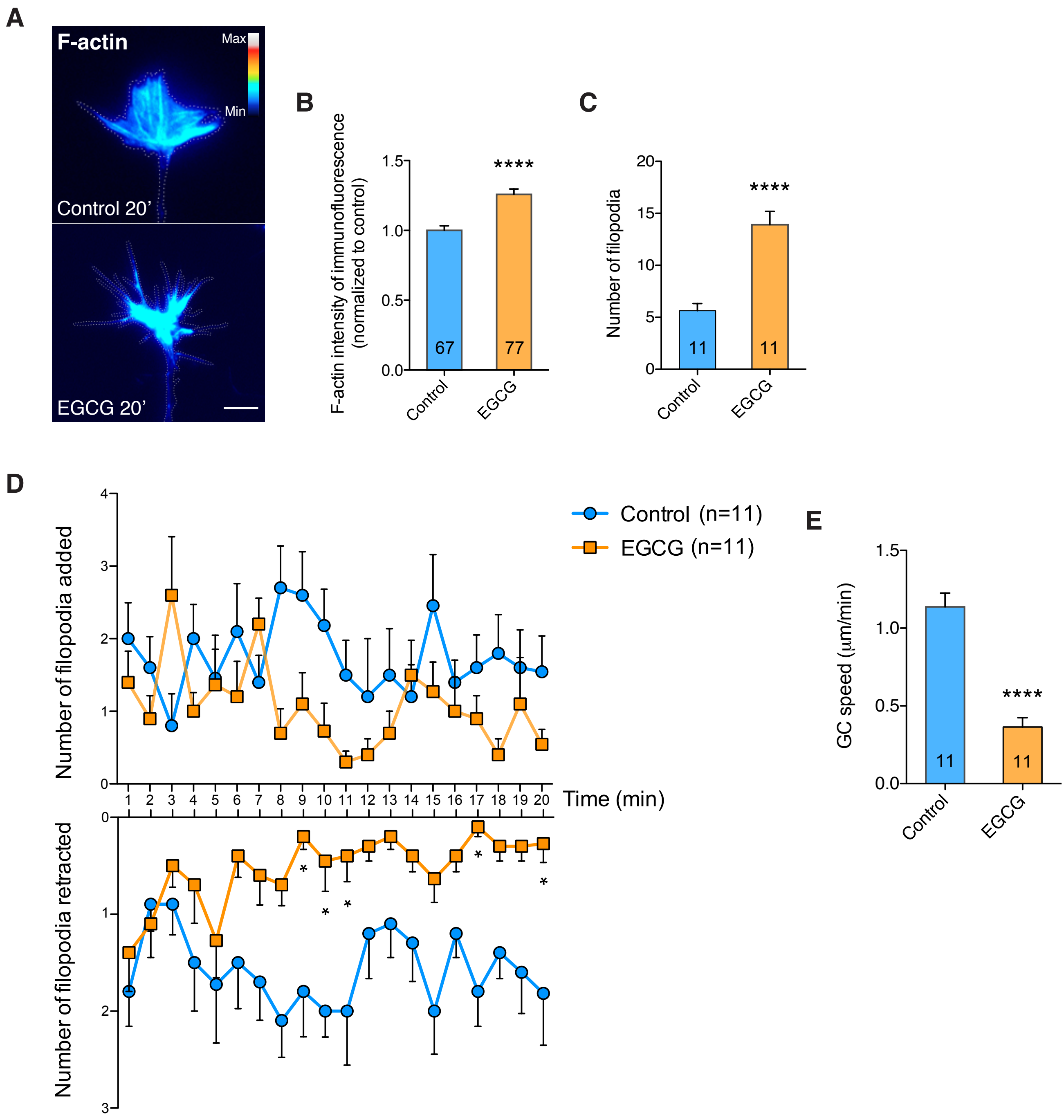

Figure S5. RpSA/67lr activation promotes increase in F-actin and cell adhesion – Related to Figure 6

(A-B) Growth cones were treated with EGCG for 20 min, stained for F-actin and IF was measured. EGCG induced an increase in F-actin signal (Unpaired t-test). **(C)** EGCG induced an increase in the number of filopodia (Paired t-test). **(D)** EGCG was added at T_0 and live imaging was carried out on growth cones. EGCG did not affect the number of filopodia added but decreased the number of filopodia retracted (Two-way ANOVA). **(E)** EGCG decreased the growth cone speed (Paired t-test). Scale bar 5 μm. Error bars s.e.m.

Supplemental Figure 6

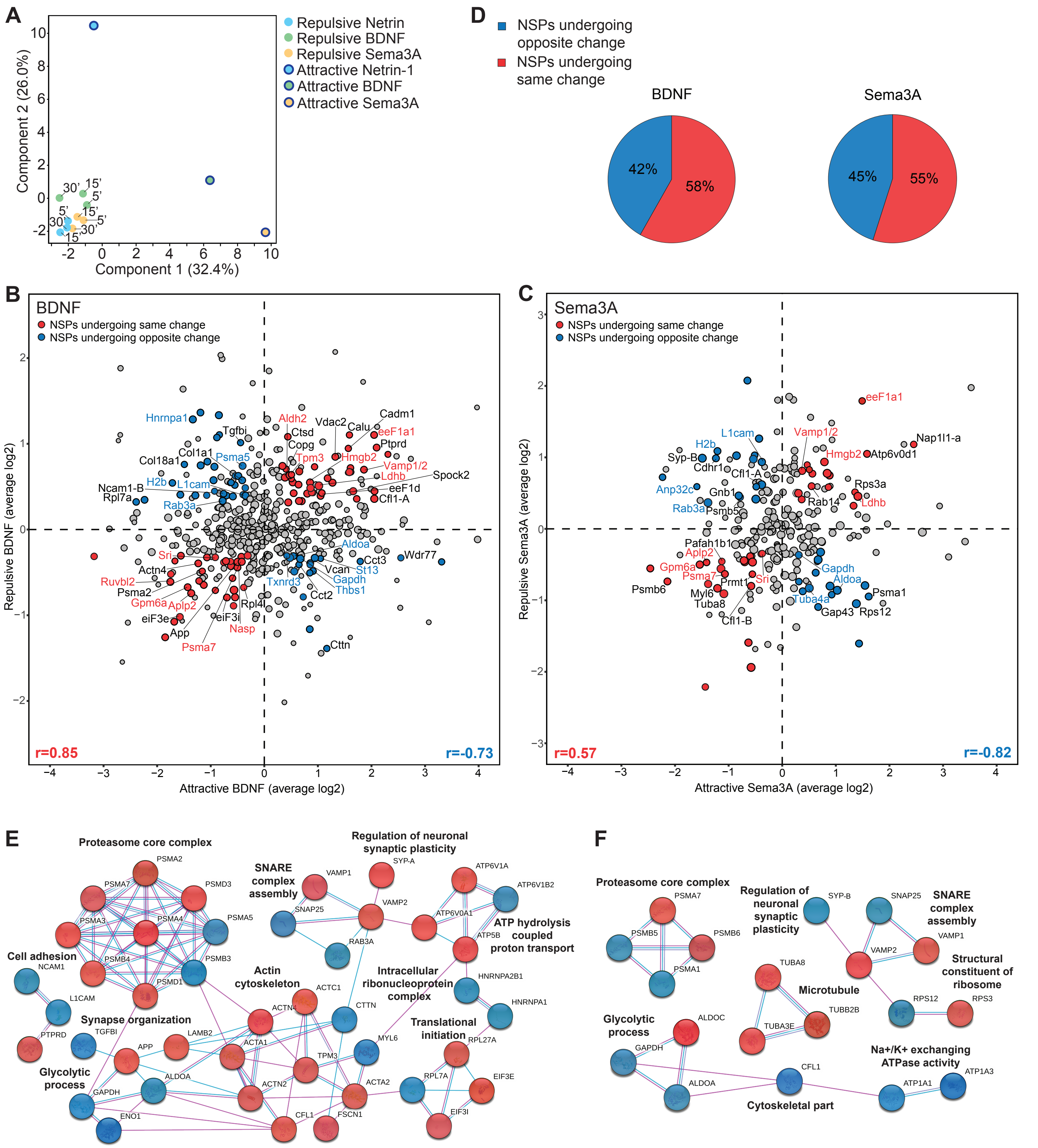

Figure S6. Analysis of the nascent axonal proteome in repulsive *vs* attractive conditions – Related to Figure 7

(A) Principal Component Analysis (PCA) based on the common subset of NSPs identified in response to both repulsive (three time points) and attractive Netrin-1, BDNF, Sema3A. Data were plotted using the first two PCs. **(B)** Repulsive and attractive BDNF ratios were plotted. Dot size correlates to count number, colored dots indicate 'commonly regulated NSPs' (count > 50%, average ratio > |0.30|). Blue indicates NSPs undergoing opposite change, red indicates NSPs undergoing same change. Examples of NSP changes are labeled with protein name (for complete list see Table S5). Specifically, protein names indicated in blue or red indicate respectively NSPs undergoing opposite or same change in response to at least two distinct attractive cue stimulations among the three investigated. **(C)** Repulsive and attractive Sema3A ratios were plotted. Dot size correlates to count number, colored dots indicate 'commonly regulated NSPs' (count > 50%, average ratio > |0.30|). Blue indicates NSPs undergoing opposite change, red indicates NSPs undergoing same change. Examples of NSP changes are labeled with protein name (for complete list see Table S5). Specifically, protein names indicated in blue or red indicate respectively NSPs undergoing opposite or same change in response to at least two distinct attractive cue stimulations among the three investigated. **(D)** Common NSP changes after converting BDNF and Sema3A repulsion into attraction (count > 50%, average ratio > |0.30|)**. (E)** Network-based cluster analysis of the enriched BDNF-induced NSP changes in common between repulsion and attraction, and their associated functional classes (p-value < 0.1). Blue nodes indicate NSPs undergoing opposite change, red nodes indicate NSPs undergoing same change, light blue lines indicate interactions known from databases, purple lines indicate interactions experimentally determined. Disconnected nodes are not shown. **(F)** Network-based cluster analysis of the enriched Sema3A-induced NSP changes in common between repulsion and attraction, and their associated functional classes (p-value < 0.1). Blue nodes indicate NSPs undergoing opposite change, red nodes indicate NSPs undergoing same change, light blue lines indicate interactions known from databases, purple lines indicate interactions experimentally determined. Disconnected nodes are not shown, i.e. more components for each enriched functional cluster have been detected (see also Figure S7).

Supplemental Figure 7

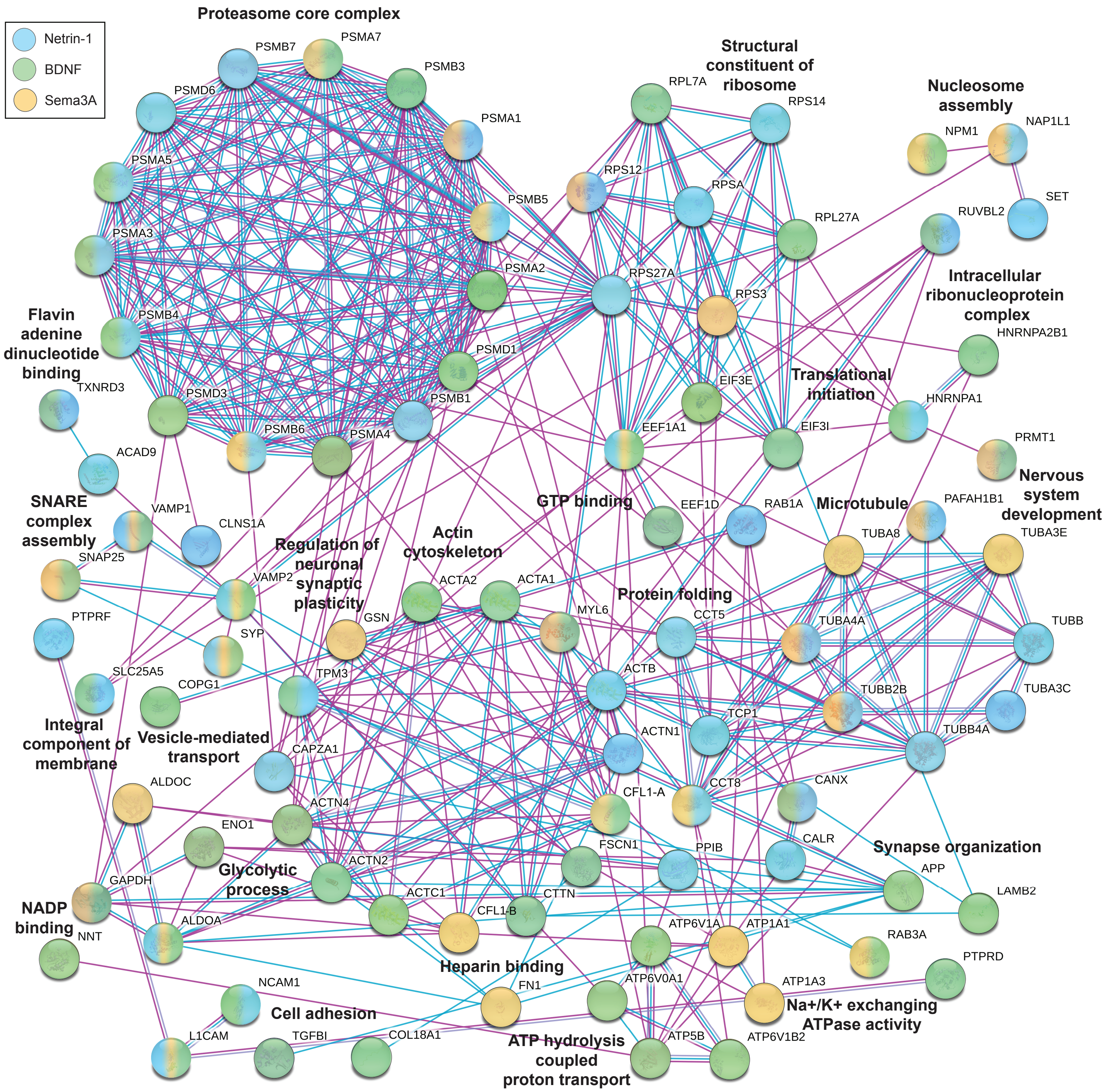

Figure S7. Network-based functional overview of cue-induced NSP changes shared between repulsion and attraction – Related to Figure 7

Network-based cluster analysis of the enriched cue-induced NSP changes in common between repulsion and attraction and their associated functional classes (p-value < 0.1). Light blue nodes indicate NSPs regulated by Netrin-1, green nodes indicate NSPs regulated by BDNF, yellow nodes indicate NSPs regulated by Sema3A, light blue lines indicate interactions known from databases, purple lines indicate interactions experimentally determined. Disconnected nodes are not shown.
